# Long-term mitigation of drought changes the functional potential and life-strategies of the forest soil microbiome involved in organic matter decomposition

**DOI:** 10.3389/fmicb.2023.1267270

**Published:** 2023-09-29

**Authors:** Martin Hartmann, Claude Herzog, Ivano Brunner, Beat Stierli, Folker Meyer, Nina Buchmann, Beat Frey

**Affiliations:** ^1^Department of Environmental Systems Science, Sustainable Agroecosystems, Institute of Agricultural Sciences, ETH Zürich, Zürich, Switzerland; ^2^Forest Soils and Biogeochemistry, Swiss Federal Research Institute WSL, Birmensdorf, Switzerland; ^3^Department of Environmental Systems Science, Grassland Sciences, Institute of Agricultural Sciences, ETH Zürich, Zürich, Switzerland; ^4^Data Science, Institute for AI in Medicine, University Hospital Essen, University of Duisburg-Essen, Essen, Germany; ^5^Argonne National Laboratory, Argonne, IL, United States; ^6^Computation Institute, University of Chicago, Chicago, IL, United States; ^7^Department of Medicine, University of Chicago, Chicago, IL, United States

**Keywords:** climate change, drought, forest, soil microbiome, decomposition, metagenomics, metabarcoding, stable isotope probing

## Abstract

Climate change can alter the flow of nutrients and energy through terrestrial ecosystems. Using an inverse climate change field experiment in the central European Alps, we explored how long-term irrigation of a naturally drought-stressed pine forest altered the metabolic potential of the soil microbiome and its ability to decompose lignocellulolytic compounds as a critical ecosystem function. Drought mitigation by a decade of irrigation stimulated profound changes in the functional capacity encoded in the soil microbiome, revealing alterations in carbon and nitrogen metabolism as well as regulatory processes protecting microorganisms from starvation and desiccation. Despite the structural and functional shifts from oligotrophic to copiotrophic microbial lifestyles under irrigation and the observation that different microbial taxa were involved in the degradation of cellulose and lignin as determined by a time-series stable-isotope probing incubation experiment with ^13^C-labeled substrates, degradation rates of these compounds were not affected by different water availabilities. These findings provide new insights into the impact of precipitation changes on the soil microbiome and associated ecosystem functioning in a drought-prone pine forest and will help to improve our understanding of alterations in biogeochemical cycling under a changing climate.

## 1. Introduction

Soil microorganisms play a critical role in maintaining the processes that underpin the stability of ecosystem functions, including plant growth, decomposition, nutrient cycling, and carbon (C) sequestration (Bardgett and van der Putten, 2014; Baldrian et al., 2023). Decomposition of organic matter is fundamental to the productivity of forest ecosystems and determined mainly by litter quality, climatic conditions, and structure and function of the decomposer community (Prescott, 2010). Water availability can affect decomposition directly by influencing organic matter fragmentation and indirectly through altering microbial activity and community composition (Yahdjian et al., 2006; Hartmann et al., 2017; Herzog et al., 2019). Recurring drought can alter soil bacterial and fungal communities in temperate forest soils (Preece et al., 2019). A decline in primary production under long-term drought and the associated reduction in fresh C inputs have been shown to induce shifts from copiotrophic to metabolically more versatile oligotrophic communities (Hartmann et al., 2017), which could ultimately alter the decomposition of aboveground inputs and root litter that is largely composed of cellulose and lignin. While cellulose has a rather simple structure, which most bacteria and fungi are able to rapidly cleave using cellulases (Bhat and Bhat, 1997), lignin degradation requires specialized and exclusive enzymes such as peroxidases or laccases (Abdel-Hamid et al., 2013). Consequently, it is postulated that cellulose is degraded at a higher rate than lignin (Berg and McClaugherty, 2008), and specialized lignin degraders such as fungi from the order Agaricomycetes evolved to fill the niche on lignin-rich plant litter (Floudas et al., 2012). Thus, the readily degradable cellulose might be preferentially degraded by a microbial community dominated by copiotrophic species, whereas the persistent lignin would be degraded only by a specialized community dominated by oligotrophic species (Cleveland et al., 2014).

The fate of lignin in soil, however, is still poorly understood when compared to cellulose (Baldrian and Valaskova, 2008; López-Mondéjar et al., 2016). In recent years, it has become evident that not only fungi but also many bacteria can partially degrade lignin (López-Mondéjar et al., 2019; Atiwesh et al., 2022). Certain taxa frequently co-occur during the degradation process, possibly indicating mutualistic interactions (Wilhelm et al., 2021), but non-mutualistic interactions in which secondary consumers either benefit from the enzyme production of the primary degraders (Voříšková and Baldrian, 2013) or directly feed on the biomass of these primary degraders (Brabcová et al., 2016) are also common. However, few studies have analysed the succession of complex microbial communities decomposing plant-derived C compounds by harnessing the potential of high-throughput DNA sequencing techniques (Herzog et al., 2019; Kohout et al., 2021) and the combination with stable isotope tracers (Haichar et al., 2007; Pepe-Ranney et al., 2016; Heijboer et al., 2018). The combination of DNA stable isotope probing (DNA-SIP) with high-throughput DNA sequencing has proven to be a robust, integrating approach to identify microbial members actively involved in the consumption of a given substrate (Dumont and Murrell, 2005). This promising technique was successfully applied to study C cycling in different terrestrial environments such as cropland (Lee et al., 2011; Kramer et al., 2016), forests (Wilhelm et al., 2017, 2019), glacier fore-fields (Rime et al., 2016), and arctic tundra (Deslippe et al., 2015; Tao et al., 2020). Importantly, adding labelled substrate might not only reveal active degraders, but also trace the C flow through the microbial food web into secondary consumers (so called cross-feeders), although distinguishing between the two remains challenging.

In addition, deep sequencing approaches such as shotgun metagenomics offer a broad-scale assessment of the functional potential encoded in the resident microbial community, specifically the potential for catabolic degradation of plant components such as lignin and cellulose (Wilhelm et al., 2017, 2019; Levy-Booth et al., 2021). Since these genes may not be transcribed under the given conditions, an additional direct assessment of the active microbial community and their function is crucial (Bastida et al., 2017). One prospecting method to identify the active decomposer community is the analysis of expressed genes encoding for enzymes involved in cellulose or lignin degradation, e.g., cellulases, peroxidases, laccases (Žifčáková et al., 2017). However, such metatranscriptomics surveys are limited by the time scale covered, as microbial gene expression can vary within minutes. Therefore, the combination of shotgun metagenomics to assess long-term shifts in the metabolic potential of the soil microbiome and SIP-based metabarcoding to investigate the active C-degraders represents a promising toolbox to better understand the response of microbial decomposers to climate change factors. Such knowledge is important to anticipate terrestrial C fluxes and feedback mechanisms under future climates (Crowther et al., 2016; Kowalska and Grobelak, 2020).

In the present study, overall shifts in microbial functional potential (shotgun metagenomes) between naturally drought-affected and long-term irrigated forest soils were assessed and coupled to changes in microbial lignin and cellulose degrading communities (DNA-SIP metabarcoding) using soil incubations. For this purpose, ^13^C-labeled lignin and cellulose extracted from maize were amended to soil microcosms to identify bacterial and fungal taxa involved in their decomposition. We hypothesized that precipitation changes alter the relative abundance of genes involved in C and nutrient cycling as well as stress response mechanisms. Specifically, we hypothesized that genes involved in nitrogen and phosphorus cycling are more prevalent in irrigated soils that are supplied with higher amounts of fresh C inputs (N and P-limited), whereas genes involved in C cycling are more prevalent in dry soils where only recalcitrant C compounds are left. Genes involved in mechanisms protecting against stresses like desiccation and starvation (e.g., different cell walls, spore formation, dormancy) should increase in water-limited soils. We further hypothesized that different microbial communities are involved in cellulose and lignin degradation in irrigated and dry soils and that this translates into different rates of degradation of these compounds. To address these hypotheses, we made use of a unique long-term irrigation experiment installed in a drought-prone Scots pine forest ecosystem in the central European Alps, where irrigation since 2003 has aimed to alleviate drought stress on pine trees caused by natural, prolonged and more frequent summer droughts (Dobbertin et al., 2010).

## 2. Materials and methods

### 2.1. Experimental system and soil sampling

In the mature drought-prone Scots pine (*Pinus sylvestris*) forest stand Pfynwald in Valais, Switzerland (46°18’N, 7°37′E, 615 m.a.s.l.) with pines having an average age of about 100 years, eight plots (25 × 40 m each) were installed, four of which have been irrigated during the summer months (April–October) since 2003, which are further termed “irrigated,” and four act as water-limited controls, which are further termed “dry.” More details about the setup have been published previously (Herzog et al., 2014). The site naturally receives around 520 mm precipitation per year. In the irrigated plots, trees were supplied with about twice the annual precipitation using an automated sprinkler system, resulting in about 1,100 mm per year (Herzog et al., 2014; Hartmann et al., 2017). This resulted in an yearly average water content of 27.8 ± 0.7% in the dry plots and 34.3 ± 0.6% in the irrigated plots measured by time domain reflectometry (Herzog et al., 2014). Soil samples for shotgun metagenome sequencing were sampled in October 2012 at the end of the irrigation period with a quantitative soil pit approach using a metal frame of 20 × 20 cm as previously described (Hartmann et al., 2017). In brief, four soil samples were collected at four different locations in each of the eight plots (at least 5 m distance from the plot edge) at three soil depths, i.e., the organic F-horizon as well as at 0–2 cm and 5–10 cm depths of the mineral soil. The four individual replicates per plot were pooled for each plot separately, resulting in four biological replications (=plots) per treatment and soil layer (2 treatments × 4 replicates × 3 depths = 24 samples). The collected soil samples differed in soil water content by an average of 2.5% between the dry and irrigated plots. The fresh soil samples were homogenized using a 2-mm sieve, and then immediately frozen and kept at −80°C until further processing. Soil samples for the SIP microcosm experiment were collected from the topsoil (0–10 cm depth) in November 2016. From each of the eight plots, 2 × 10 cm^3^ soil samples were taken with a spade and stored at 4°C.

### 2.2. Shotgun metagenome sequencing

Nucleic acids were extracted from 0.25 g soils using a bead-beating procedure as described previously (Frey et al., 2006). DNA concentrations were determined using PicoGreen (Molecular Probes, Eugene, OR, USA). A previous metabarcoding survey of the bacterial and fungal communities in the collected 24 samples (2 treatments × 3 depths × 4 replicated plots) showed significant effects of both irrigation and depth on the communities, but no interaction effects between irrigation and depth (Hartmann et al., 2017). Therefore, since the treatment effects were not dependent on the depth, the DNA extracts obtained from the two different mineral soil horizons of each replicate were equimolarly pooled prior to deep shotgun sequencing, leaving four biological replicates (=4 plots) per treatment (=8 DNA samples in total). The F-horizon previously examined by metabarcoding was not included in the analysis. DNA extracts were sent to the Génome Québec Innovation Center at McGill University (Montreal Canada) for library preparation using the NEBNext® Ultra II DNA Library Prep Kit for Illumina® (New England Biolabs, Ipswich, MA, USA) and 125 bp paired-end sequencing on the Illumina HiSeq 2,500 (Illumina, San Diego, CA, USA), yielding a total of 272,308,159 paired-end reads.

Functional annotation of the sequences was performed once based on the short reads (without assembly) and once based on assembled contigs. This was done because the two approaches might provide different views on the dataset. On the one hand, annotation of genes will likely be more accurate using longer reads (contigs), with the short reads potentially providing insufficient annotation or even overprediction (Tamames et al., 2019). On the other hand, only a fraction of the reads mapped back to the assembled contigs (i.e., 18% of the annotated short reads in this study), providing a narrower window on the gene composition compared to what was obtained via short read annotation. Furthermore, the validity of contigs obviously depends on assembly quality, potentially generating artefactual hybrid sequences (Dong and Strous, 2019).

For the short reads, raw sequences were uploaded to MG-RAST (Meyer et al., 2008) and processed using the default settings for data curation, removal of artificial duplicate reads, gene calling and feature annotation. Annotation was performed against M5nr (Wilke et al., 2012) and assignments were made at an e-value of 1e^−5^, identity of 60%, and a minimal alignment length of 30. A total of 96,446,879 sequences were assigned to the SEED Subsystem classification system (Overbeek et al., 2013). Subsystems are collection of proteins that are grouped by their relationship with respect to a specific function (Overbeek et al., 2005). For the assembled contigs, the following customized pipeline was used. Adapters were trimmed from raw reads using CUTADAPT v1.16 (Martin, 2011) and exact duplicate reads were removed using PRINSEQ v.20.4 (Schmieder and Edwards, 2011). Low quality reads were removed using the fastq_filter function (Edgar and Flyvbjerg, 2015) implemented in USEARCH v9.2.64 (Edgar, 2010). Digital normalization was performed on the quality filtered reads using BIGNORM (Wedemeyer et al., 2017). Assembly was done using MEGAHIT (Li et al., 2015b) with the meta-large kmer parameter setting. Quality filtered short reads were mapped onto the resulting contigs using the default settings of BOWTIE2 (Langmead and Salzberg, 2012) in order to get estimates of read counts per contig. Annotation of the contigs was done again using MG-RAST against M5nr with the threshold levels described above and a total of 17,288,988 sequences were assigned to the SEED Subsystem. Identification of genes encoding for carbohydrate-active enzymes (CAZymes) was done by running CAZymes-family specific hidden Markov models (HMM v8) from dbCAN2 (Zhang et al., 2018) based on the CAZy database (Lombard et al., 2013) against the predicted proteins retrieved from MG-RAST using HMMER v3.3 (Eddy, 2008). An e-value < 1e^−10^ and coverage > 0.35 was used as cut-off for the contigs, whereas the same e-value but no coverage cut-off was applied for the short reads due to the short length of the predicted proteins. These annotations yielded a total of 4,553,856 sequences (11% of the total predicted proteins) being assigned to 298 CAZymes families for the contigs, and 1,195,722 sequences (0.3%) being assigned to 364 CAZymes families for the short reads. The following detected CAZymes families were considered to be involved in lignocellulose degradation: auxiliary activities (AA) families 1, 2, 3, 4, 7, 9, 10; carbohydrate−binding module (CBM) families 2, 4, 6, 8, 11, 16, 44, 46, 63; and glycoside hydrolases (GH) families 1, 3, 4, 5, 6, 8, 9, 10, 12, 30, 44, 45, 48, 51, 74, 94, 116.

### 2.3. Microcosm experiment

Soil microcosms were set up in a climate chamber. The stable isotope probing (SIP) study was designed similar to Rime et al. (2016). Three grams of fresh sieved (2 mm) and mixed soil (C concentration 13–19%; Hartmann et al., 2017) from each of the eight plots from Pfynwald was weighed into 100 ml Polypropylene screw lid cups (Sarstedt, Newton, NC, USA). After 24 h of equilibration, the cups were closed with perforated screw lids. Four different C sources, extracted from maize leaves (*Zea mays*), were used as external C source and were added to the soils in the cups: non-labelled cellulose, ^13^C-labeled cellulose, non-labelled “Klason” lignin, and ^13^C-labeled “Klason” lignin. Soils without added C served as control (No C). In total, 160 cups were set-up with five C sources (^13^C “Klason” lignin, ^13^C cellulose, non-labelled ^12^C “Klason” lignin, non-labelled ^12^C cellulose, No C), soils from eight plots (four non-irrigated and four irrigated plots), and four replicates per C source and plot. For the non-labelled maize material (henceforth termed “^12^C”: 98.9 atom% ^12^C, 1.1 atom% ^13^C), corn cobs were purchased in a grocery, the leaves (“husks”) detached from the corn cobs, and cellulose and lignin extracted from the leaves and milled as described previously (Herzog et al., 2014). The labelled ^13^C-cellulose and ^13^C-lignin (henceforth termed “^13^C”: >97 atom% ^13^C) from maize leaves was purchased from IsoLife (IsoLife, Wageningen, The Netherlands) who used similar extraction techniques (personal communication). It must be noted here that these extracted materials might still contain impurities (e.g., hemicellulose, aromatics) that cannot be entirely removed during extraction. A total of 25 mg of each powdered C source material was added individually to the soil (only one C source per cup) and mixed in, resulting in an approximate ^13^C addition of 12 mg for cellulose and 18 mg for lignin, which corresponds to approximately 3% of the total C content of the soils per cups. To control the influence of the soil mixing, the No C control was also mixed. The soil water content was quantified for the soils from the dry (29%) and irrigated (33%) plots and standardized to 30% (of soil mass) and adjusted every second day, using distilled water. By keeping the soil moisture content constant between the two treatments, the experiment focused on the legacy effect of an altered soil microbiome on decomposition. The cups were placed in a dark climate chamber and remained at constant 20°C to simulate the mean summer temperature (June to September) at Pfynwald.

### 2.4. CO_2_ measurements and ^13^C signature in respiration

To measure the gas flux, the cups were put in air-tight 1 L glass jars with a lid containing a septum enabling gas sampling with a syringe. The flux was measured three times during 24 h, at days 0, 1, 2, 4, 6, 8, 10, 16, and 28. For each sampling event, three 1 ml samples were taken each at 0, 12, and 24 h after lid closing. Each 1 ml sample was then released into an Exetainer vial (Labco Limited, Lampeter, United Kingdom) and supplemented with 12 ml of laboratory air leading to a dilution of 1:12. The CO_2_ concentrations and their ^13^C signature were measured with a Trace GC Ultra gas chromatograph (Thermo Fisher Scientific, Waltham, MA, USA) coupled with a Delta V Advantage isotope-ratio mass spectrometer (Thermo Fisher Scientific). CO_2_ rates were calculated using a linear regression over the three sampling points during the 24 h period. Due to potential contamination of the Exetainers with too high CO_2_ concentrations, two outliers were removed. δ^13^C values were expressed as per mil (‰). in relation to the Vienna-Pee Dee Belemnite gauged reference materials with a measurement precision of ±0.20%.

### 2.5. DNA extraction and fractionation

Consecutive sampling of soil for DNA-SIP analysis was performed at days 2, 4, 8, and 28. Per sampling day and cup, a soil sample was taken with an ethanol sterilized spatula, put into an Eppendorf tube, and then stored in a freezer at −80°C until DNA extraction. DNA was extracted from 0.25 g of frozen soil using the Power Soil DNA isolation kit (MoBio Laboratories, Carlsbad, CA, USA). DNA concentrations were measured by PicoGreen fluorescence spectroscopy. Enriched ^13^C-DNA was isolated after ultracentrifugation in a CsCl gradient (Neufeld et al., 2007) according to protocols previously established (Rime et al., 2016). In brief, approximately 5 μg of DNA were dissolved in 4.8 ml CsCl buffer. Optical density of the CsCl was adjusted to 1.4029 ± 0.0002 with a Refracto 30PX refractometer (Mettler-Toledo, Columbus, OH, USA) corresponding to the final concentration of 1.723 g ml^−1^. Samples were filled into ½x2 polyallomer tubes (Beckman Coulter, Brea, CA, USA) and sealed following manufacturer instructions. For ultracentrifugation, a Vti-65.2 vertical rotor in an optima TM L-80 XP ultracentrifuge (Beckman Coulter) was used for 40 h at 177000 g. In total, 18 fractions, of approximately 250 μl gradient solution each were collected dropwise from the tube bottom after a small hole was carefully punctured into the top of the tubes to let air enter. Optical density of the fractions was measured with the Refracto 30PX, for checking the formation of the gradient. A subset of the samples was used to correlate density with DNA concentration measured by PicoGreen. Fractions 10 + 11 (counted from the tube bottom) with a density of 1.72–1.74 g ml^−1^ CsCl were defined as the heavy fraction, and fractions 15 + 16 (counted from the tube bottom) with a density of 1.70–1.71 g ml^−1^ CsCl were defined as the light fraction, as described earlier (Wang et al., 2015; Rime et al., 2016). The DNA in these fractions was precipitated with 1.2 ml polyethylene glycol buffer (30% PEG6000 and 1.5 M NaCl, Sigma Aldrich, St. Louis, MO, USA) by centrifugation (16,000 *g*, 30 min), washed with 150 μl 70% ethanol, and eluted in 30 μl AE buffer (Qiagen, Hilden, Germany). Fractions defined as heavy fraction and fractions defined as light fraction were pooled, respectively, yielding a total 320 samples, i.e., 5 C sources (^13^C-cellulose, non-labeled cellulose, ^13^C-lignin, non-labeled lignin, no C) × 2 treatments (dry, irrigated) × 4 field replicates × 4 incubation time points (2, 4, 8, 28 days) × 2 fractions (heavy, light).

### 2.6. Amplicon sequencing of SIP fractions

The DNA in the SIP fractions was quantified with PicoGreen, and 10 ng of template DNA was used for PCR amplification, targeting the ribosomal small-subunit RNA genes (region V3-V4) for bacteria using primers 341F and 806R (Frey et al., 2016), and the internal transcribed spacers 2 (ITS2) for fungi using primers ITS3ngs and ITS4ngs (Tedersoo and Lindahl, 2016) as previously described (Frey et al., 2016). Sequencing of the PCR products was performed at the Génome Québec Innovation Center at McGill University (Montréal, Canada). Barcoding was done using the Fluidigm Access Array technology (Fluidigm, South San Francisco, CA, USA) and paired-end sequencing on the Illumina MiSeq v3 platform (Illumina Inc., San Diego, CA, USA), yielding a total of 55,523,325 bacterial and 31,000,380 fungal reads, respectively. Sequences were processed using a customized bioinformatics pipeline largely based on VSEARCH v2.8 (Rognes et al., 2016) as described in detail previously (Herzog et al., 2019). In brief, steps included paired-end read merging, contaminant removal, primer trimming, quality filtering, delineation into amplicon sequence variants (ASVs), chimera removal, target verification and taxonomic classification. Taxonomic classification of bacterial and fungal reads was performed using the SILVA v.132 (Pruesse et al., 2007) and UNITE v.80 (Abarenkov et al., 2010) databases, respectively. After bioinformatic processing, a total of 12,770,098 (39,906 ± 12,048) bacterial and 14,608,342 (45,651 ± 11,704) fungal sequences remained for the 320 samples.

### 2.7. Statistics

All statistical analyses were done in R v.4.1.2 (R Core Team, 2014). The following statistical tests were carried out for the soil metagenome data. Differences in gene composition across the different samples were examined based on Bray-Curtis dissimilarities calculated from normalized and square root-transformed read count data using the function *vegdist* in the R package vegan 2.5–7 (Oksanen et al., 2016). Compositional differences were quantified using multivariate permutational analysis of variance (PERMANOVA; Anderson, 2001) implemented as the *adonis* function in vegan and visualized using principal coordinate analysis (PCoA) calculated by the *cmdscale* function of the stats v.4.1.2 package in R. Influence of multivariate dispersion was assessed using permutational analysis of variance (PERMDISP; Anderson et al., 2006) implemented as the *betadisper* function in vegan. Shannon diversity indices of the gene compositions were calculated as the mean from iteratively (100-fold) subsampled count data and using the *diversity* function in vegan. Significant differences in Shannon diversity were examined using univariate PERMANOVA and PERMDISP based on Euclidean distances with the *adonis* and *betadisper* functions in vegan. Annotated functions were classified at different functional levels using the SEED Subsystems hierarchy (Overbeek et al., 2013) by MG-RAST. Differences in relative counts of the individual categories were assessed at all hierarchical levels using ANOVA (function *aov*) in R followed by adjustment for multiple testing using the false discovery rate correction according to Storey (2002) using the R package qvalue (Storey et al., 2015). Assumptions with respect to normality and homogeneity of variance were assessed using the Shapiro–Wilk test of normality (function *shapiro.test* in R) and the Levene’s test for homogeneity of variance (function *leveneTest*, R package car v.3.0.12), respectively. The same statistical analyses were performed for the relative counts of CAZymes families.

The following statistical tests were carried out for the dataset from the SIP mesocosm study. Differences between gas measurements were assessed by multifactorial ANOVA and rank transformation was used if normality (Shapiro–Wilk test) or homogeneity of variance across groups (Levene’s test) did not apply. Tukey multiple comparisons test of means between the groups were tested using Tukey’s HSD method (function *TukeyHSD* in R). For the microbial data, low coverage samples with less than 10,000 reads as well as rare (relative abundance of less than 0.001%) and sparse (occurring in less than 3 samples) ASVs were discarded, yielding 310 bacterial and 314 fungal samples with 14,462 and 2,218 ASVs, respectively. Datasets were then split by C source, i.e., lignin and cellulose, and analysed separately. Differences in β-diversity were examined based on Bray-Curtis dissimilarities calculated from normalized and square-root transformed (aka Hellinger transformation) ASV count data using the *vegdist* function in vegan. The influence of the different experimental factors, i.e., irrigation, field plot, incubation time, C source, isotope label, and fraction, on the community structure was examined using PERMANOVA and PCoA. Constrained ordination was performed using canonical analysis of principal coordinates (CAP; Anderson and Willis, 2003) implemented as the *CAPdiscrim* function in the R package BiodiversityR v.2.14–1 (Kindt and Coe, 2005). ASVs significantly associated with the different incubation treatments (substrate × label × fraction, further called “responsive ASVs”) and significantly enriched in the heavy-labelled SIP fraction (further called “enriched ASVs”) were obtained using correlation-based indicator species analysis (De Cáceres and Legendre, 2009) using site-group combinations (De Cáceres et al., 2010) as implemented by the *multipatt* function in the R package indicspecies (De Cáceres and Legendre, 2009) followed by multiple testing correction according to Storey. Differences in community structure of the enriched ASVs was again explored by PCoA and PERMANOVA. Sankey plots generated in RAWGraphs^1^ were used to display taxonomic composition of the significantly enriched bacterial and fungal taxa.

## 3. Results

### 3.1. Shifts in the functional potential of the soil microbiome

**Figure 1:**
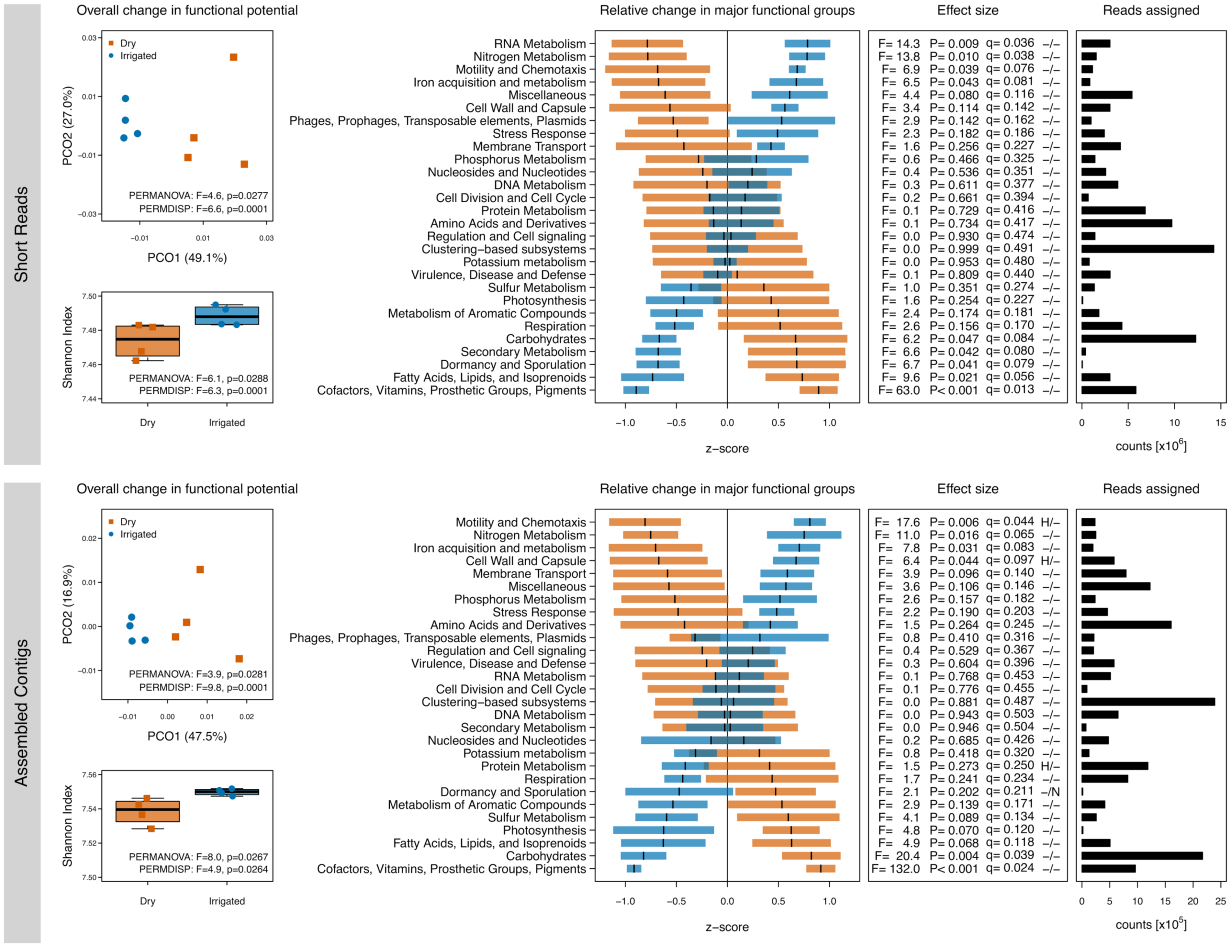
Gene diversity and compositional shifts of the soil metagenomes based on annotations to the SEED Subsystems database. Left: Shannon diversity and principal coordinate ordinations (PCOs) based on the inventory of annotated genes based on short reads (top) and assembled contigs (bottom) from dry (red) and irrigated (blue) forest soils. The variance explained by each PCO axis are provided in parentheses. Right: Relative changes of SEED Subsystems categories (level 1) in dry (red) and irrigated (blue) plots. The horizontal bars represent z-scores (means and standard errors) of relative gene abundances indicating a proportional increase or decrease of these genes in each treatment compared to the overall mean value across the entire dataset. The effect sizes of the relative changes (z-scores) due to the treatment are provided as ANOVA F-ratios, *p*-values and q-values. Positive tests for heteroscedasticity (H) and non-normality of residuals (N) are flagged. Number of assigned reads across all samples in each category are provided.

The soil metagenome analysis revealed that a decade of irrigation increased functional gene diversity (Shannon index) and shifted functional gene composition (PCoA ordination) when compared to the dry plots (Figure 1). Irrigation also reduced between-plot variability of both diversity and composition of the functional genes. Categorization of the functional annotations into SEED Subsystems revealed irrigation-dependent shifts in the relative abundance of genes involved in C and nutrient metabolism as well as genes that are linked to phenotypic responses to drought and desiccation (Figure 1), although the statistical power was limited with only four replicated forest plots. Under water-limited conditions, genes related to turnover of carbohydrates and aromatic compounds tended to be higher, whereas genes related to nitrogen (N), iron (Fe) and phosphorous (P) metabolisms tended to be higher under irrigation. Furthermore, genes related to cofactors (including many coenzymes), cell wall components (fatty acids, lipids and isoprenoids) as well as dormancy and sporulation processes tended to be higher under drought. The first level of the SEED Subsystems ontology is very broad and shifts in the underlying levels of the ontology need to be taken into account. The responses at all levels of the SEED Subsystems are provided in Supplementary Data S1 and SEED levels 1 to 3 are visualized in Supplementary Figures S2, S3 for further exploration. For example, the significant increase in genes associated with N metabolism under irrigation was primarily related to categories like nitrogen fixation, ammonia assimilation, nitrate and nitrite ammonification, and denitrification (Supplementary Figures S2, S3). Furthermore, the increase in genes related to the cell wall and capsule category under irrigation (Figure 1) was attributed to a strong increase in gram-negative cell wall components with a simultaneous relative decrease of gram-positive cell wall components (Supplementary Figures S2, S3). Notably, results based on short reads sometimes differed substantially from those based on the assembled contigs. For example, genes related to RNA metabolism were significantly higher under irrigation based on the short reads but did not show any difference based on the assembled contigs.

**Figure 2:**
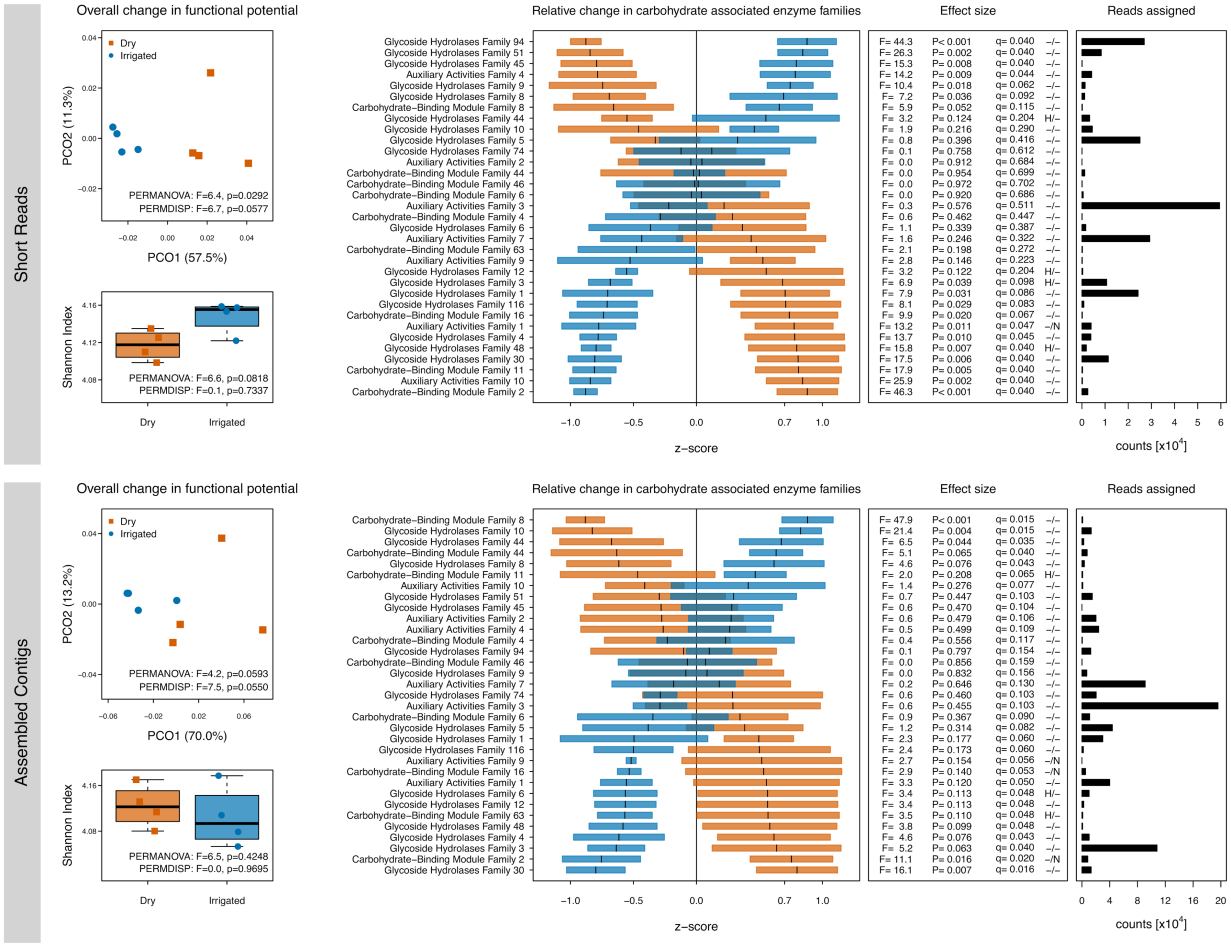
Gene diversity and compositional shifts of the soil metagenomes based on annotations to the CAZy database. Left: Shannon diversity and principal coordinate ordinations (PCOs) based on the inventory of annotated genes based on short reads (top) and assembled contigs (bottom) from dry (red) and irrigated (blue) forest soils. The variance explained by each PCO axis are provided in parentheses. Right: Relative changes of CAZy families in dry (red) and irrigated (blue) plots. The horizontal bars represent z-scores (means and standard errors) of relative gene abundances indicating a proportional increase or decrease of these genes in each treatment compared to the overall mean value across the entire dataset. The effect sizes of the relative changes (z-scores) due to the treatment are provided as ANOVA F-ratios, p-values and q-values. Positive tests for heteroscedasticity (H) and non-normality of residuals (N) are flagged. Number of assigned reads across all samples in each category are provided.

Since the C metabolism was of central interest to the study, we further explored SEED Subsystem level 2 classifications related to C metabolism (Supplementary Figure S1) as well as shifts in CAZymes families through dbCAN (Figure 2). Several irrigation-induced shifts in these functional categories were observed; however, the unassembled short reads and the assembled contigs showed again some differences. Over both datasets, genes related to di- and oligosaccharides metabolism, organic acids metabolism, and one-C metabolism tended to be higher in the dry plots (Supplementary Figure S1). Other responses were dataset-dependent, e.g., a drought-related higher number of genes related to polysaccharides metabolism and CO_2_ fixation (short read dataset) or genes related to the central carbohydrate metabolism (assembled contigs). CAZymes annotations showed irrigation-dependent responses in gene diversity and composition similar to the ones observed with the SEED subsystems annotation, although differences were less clear (Figure 2). CAZymes families involved in lignocellulose degradation (see Materials and methods) showed irrigation-dependent shifts in relative abundance, although short reads and assembled contigs showed again differences (Figure 2).

### 3.2. Soil respiration and ^13^C signatures during lignocellulose decomposition

**Figure 3:**
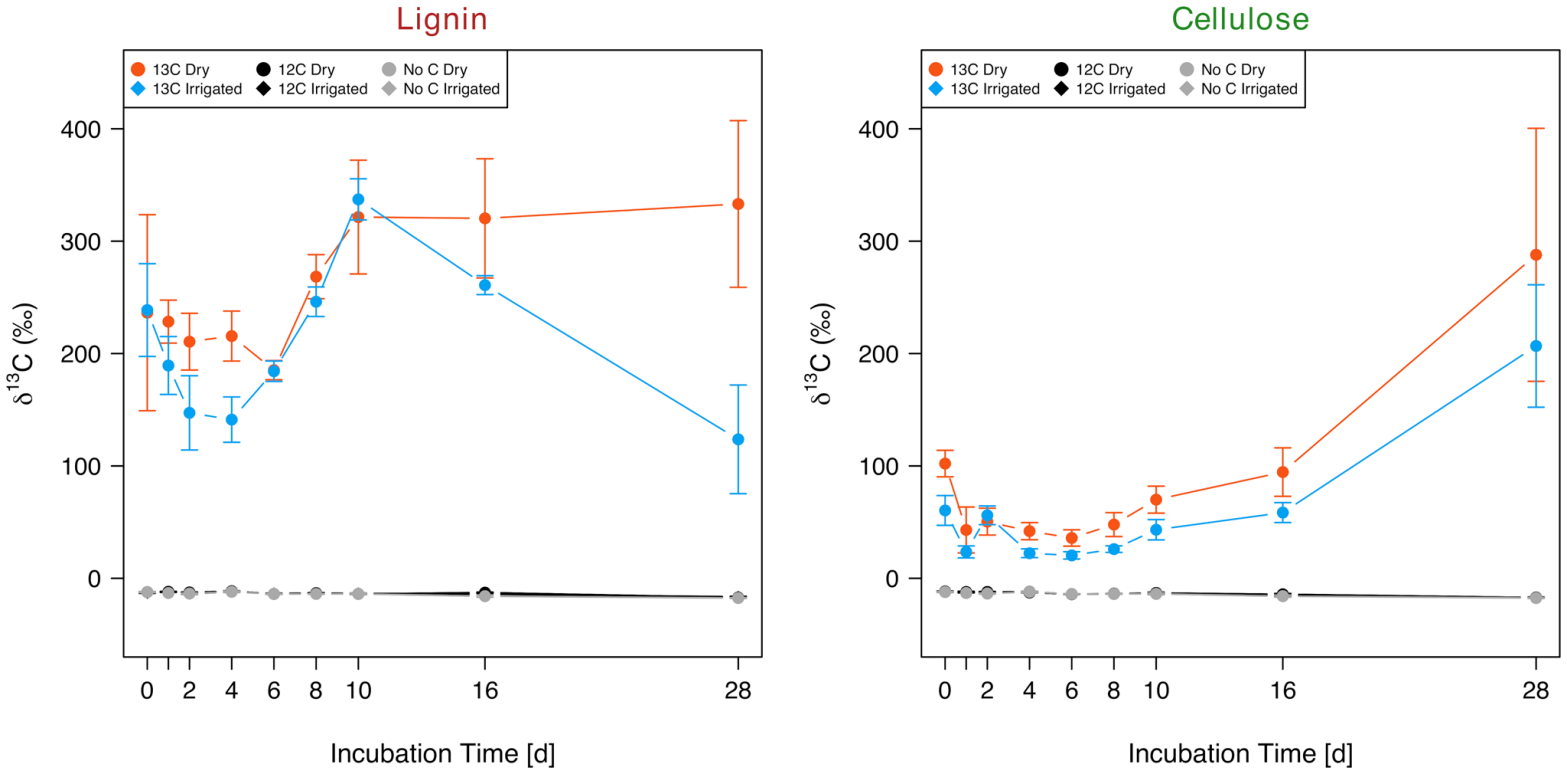
Evolution of δ^13^C in collected CO_2_ over the 28-day incubation period of two forest soils (dry vs. irrigated) supplied with ^13^C-labeled (13C) and non-labeled (12C) lignin (left) and cellulose (right), including the no substrate controls.

Production of CO_2_ in the mesocosms was neither affected by the addition of lignin nor by cellulose (Supplementary Figure S4). Nonetheless, large fluctuations in respiration rates were detected, irrespective of C addition or irrigation treatment. After incubation of the soil treated with ^13^C-labeled lignin and cellulose, the respired CO_2_ revealed a strong significant (ANOVA; *p*-value < 0.001) increase in δ^13^C (Figure 3). Lignin increased the mean δ^13^C by 252‰ in dry and 208‰ in irrigated soil compared to the non-labelled substrate addition and no-substrate addition controls. A clear drop of δ^13^C at incubation day 28 was detected in the soils from the irrigated plots compared to the soils from the dry plots. The increase in δ^13^C in respired CO_2_ was significantly higher in ^13^C-labeled lignin samples (232‰ ± 94‰) compared to ^13^C-labeled cellulose (68‰ ± 83‰; ANOVA; *p*-value < 0.001). Overall, the soils from the irrigated plots showed a tendency for a reduced ^13^C signature in the respired CO_2_ (130‰ ± 105‰) compared to the soils from the dry plots (171‰ ± 133‰) after ^13^C-labeled substrate addition (ANOVA; p-value = 0.087).

### 3.3. Microbial utilizers of lignin and cellulose

Table 1 summarizes the effects of the experimental factors on forest soil bacterial and fungal community composition during lignin and cellulose incubation. For the lignin experiment, differences in bacterial community structure were strongly driven by SIP-fractionation (Table 1, *fraction*), by the irrigation treatment history (*treatment*), and to a minor extent by incubation day (*day*), hence not by the addition of lignin (*C source*). Differences in fungal community structure were mainly driven by *treatment* and *fraction* and to a lesser extent by *C source* (lignin) and incubation *day*. For the cellulose experiment, *treatment* and *fraction* were the strongest influencing factors, whereas *C source* (cellulose) and incubation *day* had smaller effects (Table 1).

**Table 1:** Effects of the experimental factors on forest soil bacterial and fungal community structure after lignin or cellulose addition as assessed by PERMANOVA (*p*-values < 0.05 in bold).

Factor^1^	Lignin			Cellulose
	Bacteria		Fungi		Bacteria		Fungi	
	F-ratio	P-value	F-ratio	P-value	F-ratio	P-value	F-ratio	P-value
C source (C)	1.3	0.172	2.6	**0.002**	2.1	**0.023**	1.8	**0.038**
Label (L)	1.4	0.149	3.2	**<0.001**	2.0	**0.027**	2.2	**0.008**
Fraction (F)	44.3	**<0.001**	14.6	**<0.001**	48.9	**<0.001**	13.7	**<0.001**
Treatment (T)	42.9	**<0.001**	40.5	**<0.001**	45.8	**<0.001**	44.8	**<0.001**
Day (D)	2.7	**0.004**	2.4	**0.004**	2.7	**0.005**	2.8	**0.001**
Plot (P)	9.5	**<0.001**	19.8	**<0.001**	9.7	**<0.001**	20.2	**<0.001**
C × F	0.9	0.557	1.1	0.317	0.8	0.618	0.5	0.951
L × F	1.2	0.207	2.1	**0.009**	1.1	0.284	0.7	0.864
C × T	0.7	0.858	0.7	0.875	0.8	0.709	0.8	0.700
L × T	0.7	0.853	0.8	0.655	0.7	0.803	0.8	0.674
F × T	3.1	**0.002**	1.1	0.329	3.3	**<0.001**	1.2	0.229
C × D	1.1	0.320	0.8	0.632	1.1	0.298	1.0	0.414
L × D	1.1	0.320	1.3	0.139	1.3	0.179	1.3	0.150
F × D	1.4	0.128	1.3	0.187	1.4	0.125	1.5	0.104
T × D	0.8	0.643	1.1	0.348	0.9	0.501	1.0	0.426
C × P	0.6	0.939	0.6	0.925	0.6	0.871	0.7	0.813
L × P	0.6	0.905	0.5	0.982	0.6	0.881	0.7	0.837
F × P	1.2	0.248	0.6	0.944	1.2	0.268	0.4	0.999
T × P	7.6	**<0.001**	13.8	**<0.001**	7.8	**<0.001**	14.9	**<0.001**
D × P	0.5	0.964	0.5	0.965	0.7	0.833	0.7	0.842

1 Factors include C source (lignin vs. cellulose vs. no substrate), label (^13^C vs. ^12^C), fraction (heavy vs. light), treatment (dry vs. irrigated), day (2, 4, 8, 28 days) and plot (*n* = 4).

**Figure 4:**
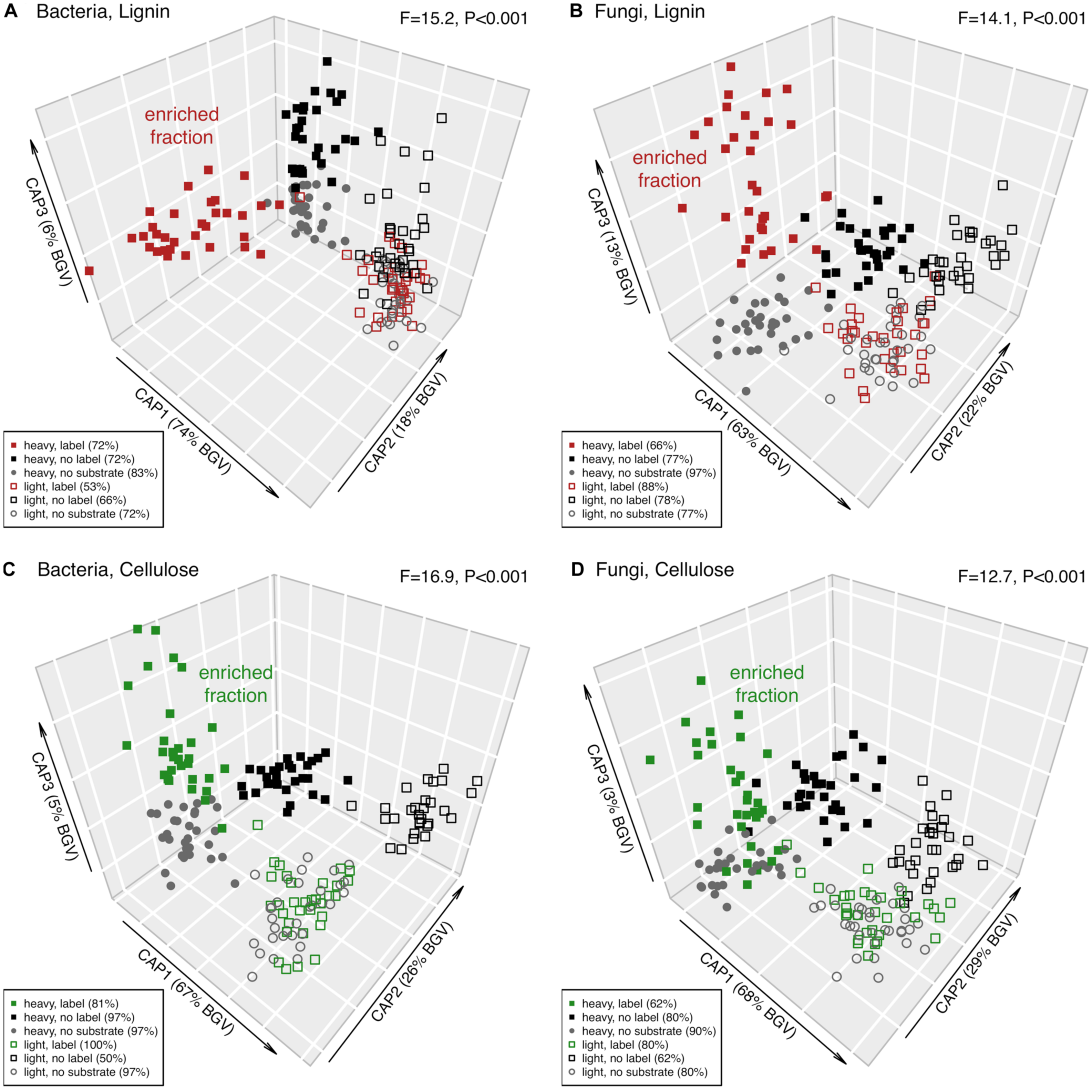
Constrained analysis of principal coordinates (CAP) of the responsive microbial community of incubated forest soils with ^13^C-labeled and non-labeled (12C) lignin and cellulose, and the no substrate control. ASVs significantly affected by the factors fraction, C source and label compile the responsive bacterial and fungal community. The amount of between group variation of each CAP axis are provided in parentheses. CAP reclassification success rates – a quantitative estimate of the degree of discrimination among the clusters achieved by the canonical axes – are provided next to the factors in the legend. Effect size (F-ratio) and level of significance (p-value) as determined by PERMANOVA testing the factor combining fraction (heavy vs. light), C source (lignin or cellulose vs. no substrate), and label (^13^C vs. ^12^C lignin or cellulose) are provided in the plot corners (see Supplementary Table S1 for details).

**Figure 5:**
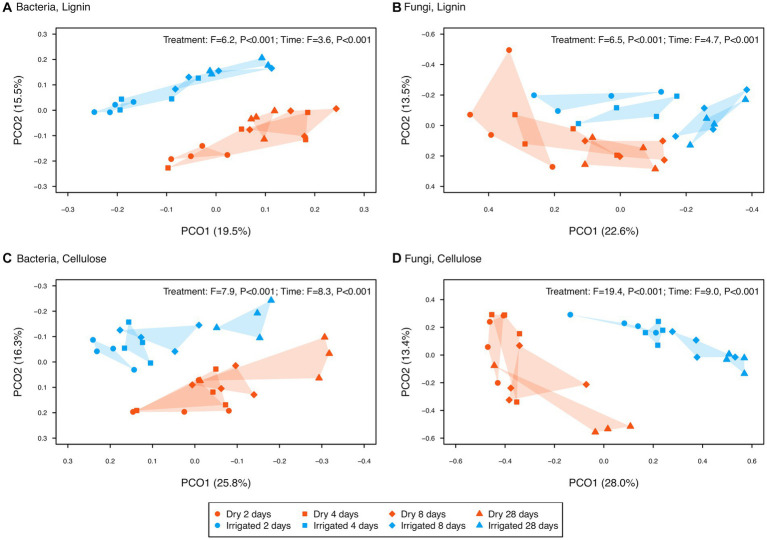
Principal coordinate ordinations (PCOs) of enriched bacterial (left panel) and fungal (right panel) ASVs in the heavy fraction extracted from two forest soils (dry vs. irrigated) incubated with ^13^C-labeled lignin (upper panel) and cellulose (lower panel) over the 28-day study period. The variance explained by each PCO axis are provided in parentheses. Effect size (F-ratio) and level of significance (*p*-value) as determined by PERMANOVA testing the factors treatment (dry vs. irrigated) and incubation time (2, 4, 8, and 28 days) are provided in the plot corners (see Supplementary Table S2 for details).

To reduce noise, ASVs not showing significant differences across the factors *C source*, *label* or *fraction* were removed, keeping only the “responsive” ASVs (ca. 67% for bacteria and ca. 8% for fungi). Based on these responsive ASVs, bacterial and fungal community structures after lignin addition revealed a clear separation of the two different density fractions (Figure 4, CAP1 axis). In the heavy fraction, the samples receiving labelled lignin formed a separate cluster (Figure 4, along CAP2 for bacteria and CAP3 for fungi). A similar separation of the labelled samples in the heavy fraction was observed after cellulose addition (Figure 4, along CAP3 for both bacteria and fungi). These heavy fraction clusters of samples supplied with ^13^C-labeled substrate (Figure 4, red and green filled symbols), here referred to as enriched microbial community, revealed a clear separation by the irrigation treatment and a successional trend from incubation day 2 to 28 (Figure 5). This significant separation by irrigation treatment and incubation time was detected for bacteria and fungi, and for lignin and cellulose additions (Figure 5).

**Figure 6:**
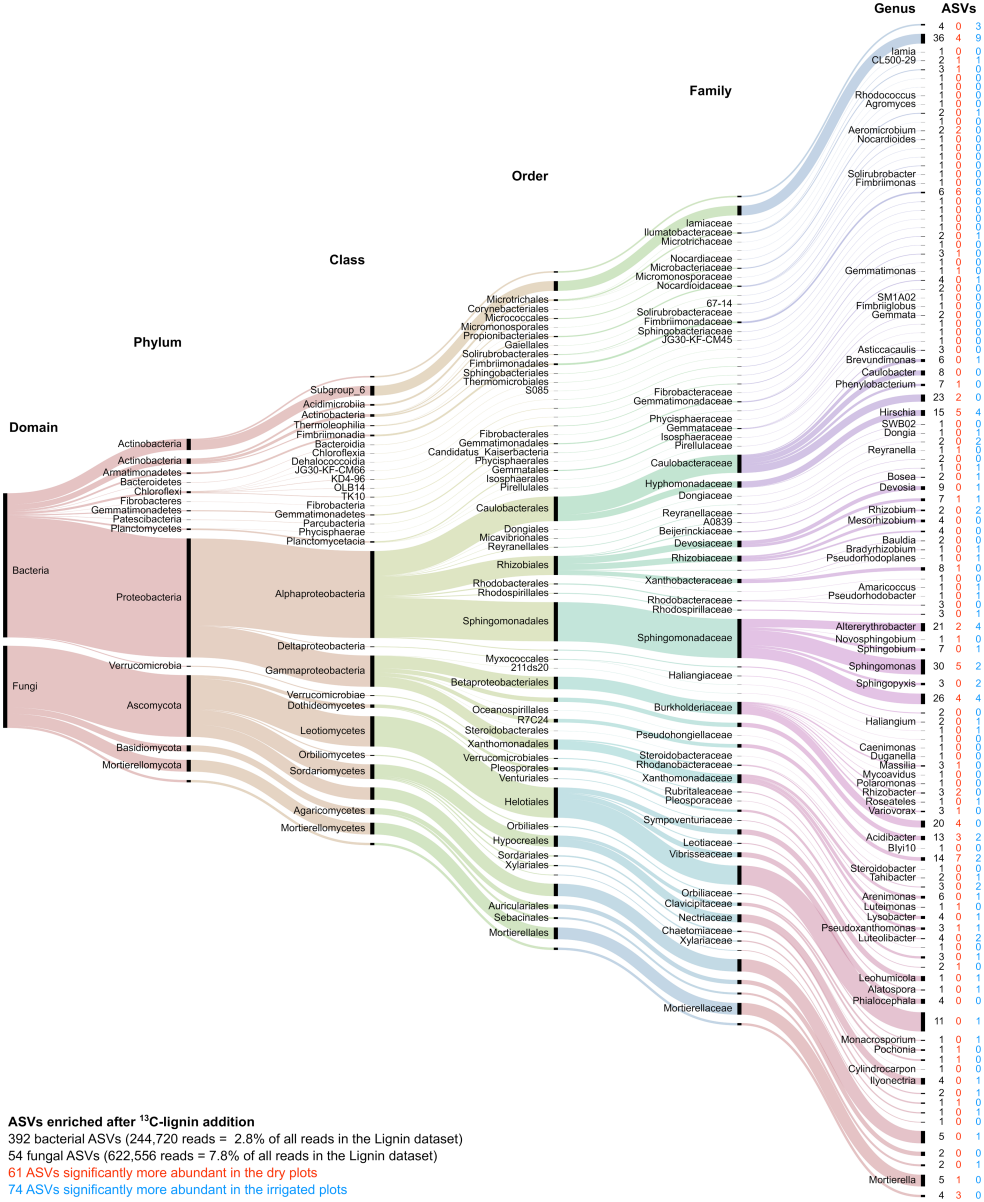
Alluvial diagram of the 446 ASVs significantly enriched after addition of ^13^C-labeled lignin. The lowest assigned taxonomic level is indicated. The size of the streams is scaled by the number of reads in each group. The number in columns indicates the number of ASVs significantly enriched by lignin addition (black) as well as those enriched ASVs that significantly increased under the dry (red) or irrigation (blue) treatments.

**Figure 7:**
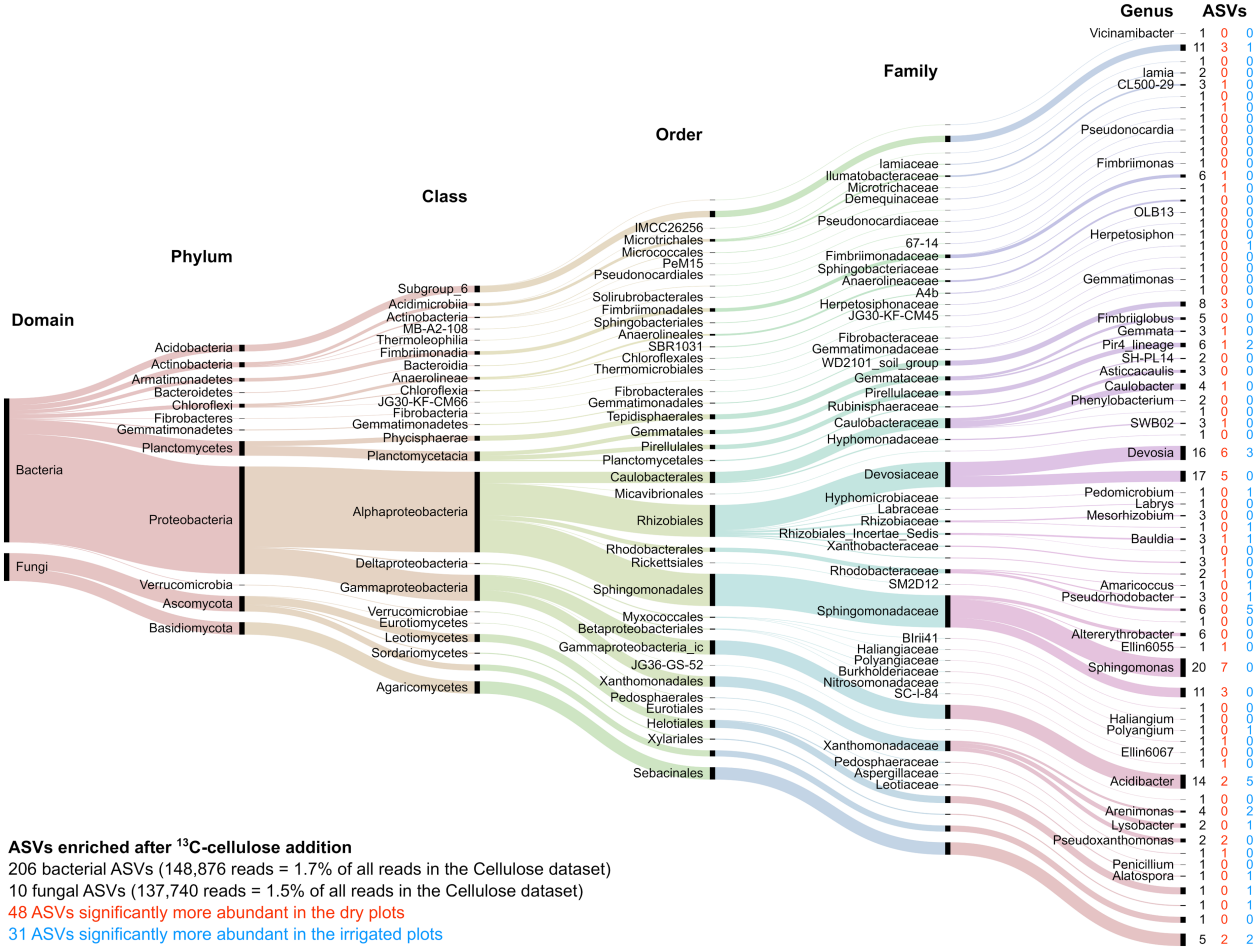
Alluvial diagram of the 216 ASVs significantly enriched after addition of ^13^C-labeled cellulose. The lowest assigned taxonomic level is indicated. The size of the streams is scaled by the number of reads in each group. The number in columns indicates the number of ASVs significantly enriched by cellulose addition (black) as well as those enriched ASVs that significantly increased under the dry (red) or irrigation (blue) treatments.

Among the “responsive” ASVs, 446 ASVs (392 bacterial and 54 fungal ASVs, respectively) have been detected that were significantly enriched in the heavy fraction after addition of ^13^C-labeled lignin, and 216 ASVs (206 bacterial and 10 fungal ASVs, respectively) were enriched after ^13^C-labeled cellulose addition. Lignin degradation, measured by lignin C incorporation, was registered for the bacterial families Sphingomonadaceae, Caulobacteraceae, Burkholderiaceae, while fungal lignin C uptake was dominated by the orders of Helotiales, Mortierellales, and Hypocreales (Figure 6). Cellulose-C was mainly taken up by the bacterial families Sphingomonadaceae, Devosiaceae, Xanthomonadaceae, and the genus Acidibacter, while the fungal orders Sebacinales and Helociales were mainly dominating the fungal cellulose uptake (Figure 7). The irrigation treatment did shift the composition of lignin degrading microorganisms, hence, often only to closely related taxa.

## 4. Discussion

### 4.1. Irrigation alters the metabolic potential of the soil microbiome

Microbes mediate numerous essential soil functions which are encoded in their collective genomes. Key functions include decomposition and transformation of organic materials and toxic compounds, nutrient and water provisioning for plants, control of pests and diseases, and regulation of greenhouse gases (Bardgett and van der Putten, 2014; Baldrian et al., 2023). These functions can be altered by management practices (Cardenas et al., 2015) or seasonal (Žifčáková et al., 2017) and climatic variations (Xue et al., 2016; Zhang et al., 2017). Organic matter decomposition and N-cycling are two of the main soil functions driven by microorganisms and have been shown to shift with temperature and water availability (Zhang et al., 2017). Here, long-term irrigation of drought-prone forest soils increased gene diversity and selected for different functional potentials related to soil C and nutrient cycling (Figures 1, 2). The relative abundance of genes related to carbohydrate metabolism (primarily one-C compounds, di- and oligosaccharides, and organic acids) was reduced in irrigated plots, while genes related to N metabolism (i.e., nitrogen fixation, ammonia assimilation, nitrate and nitrite ammonification, denitrification) and, to some degree, Fe and P metabolism increased (Figure 1; Supplementary Figure S1; Supplementary Data S1). As proposed earlier, increased primary production and higher inputs of fresh organic material via litter fall and root exudation in the long-term irrigated forest plots might have led to a shift in nutrient limitation of microbial communities from C to N (Herzog et al., 2014) and to an increased relative abundance of N scavengers (Herzog et al., 2019). The functional potential encoded in the soil metagenomes supported this hypothesis (Figure 1).

We reported previously that oligotrophic, metabolically versatile, or drought-tolerant bacterial groups increased under dry soil conditions (Hartmann et al., 2017), indicating the presence of limiting conditions due to long-term drought. This response was also mirrored in the metagenome, showing an increased relative abundance of regulatory genes encoding for cofactors (such as coenzymes), cell wall components (such as fatty acids, lipids, and isoprenoids), dormancy and sporulation under the natural dry conditions. These findings indicate microbial adaptation to increasing and prolonged droughts by shifting the species composition towards taxa with more efficient metabolism (i.e., cofactors) and protection mechanisms against desiccation and starvation (i.e., different cell walls, spore formation, dormancy) as hypothesized earlier (Hartmann et al., 2017; Herzog et al., 2019). Furthermore, the relative increase of genes encoding for gram-positive cell wall components and simultaneous decrease of genes encoding for gram-negative cell wall components under drought mirrors the strong increase of gram-positive bacteria like Actinobacteria and the strong decrease of gram-negative bacteria like Proteobacteria as reported previously (Hartmann et al., 2017). Previous assessments of soil metagenomes along resource-gradients demonstrated that increased relative abundances of genes related to carbohydrate metabolism (e.g., polysaccharides, di- and oligosaccharides and amino sugars), protein metabolism, respiration and photosynthesis are indicative of oligotrophic conditions, whereas increased abundances of genes related to RNA metabolism or motility and chemotaxis are characteristic of copiotrophic conditions (Song et al., 2017; Chen et al., 2020). These findings were largely supported by our observations (Figure 1; Supplementary Figure S1).

The potential effect of irrigation on soil C cycling was further assessed by focusing on carbohydrate-related enzymes using the CAZy database. The irrigation-driven effects on gene diversity and composition were consistent with the Subsystems-level analyses with a lesser effect size (Figure 2). Wilhelm et al. (2019) demonstrated the microbial importance for degradation of lignocellulose in forest soils. It is remarkable that not only are large differences in decay potential found across different forest sites (Wilhelm et al., 2019), but apparently also within a single forest site undergoing an irrigation treatment as in this study. Overall, the shift in relative abundances of carbohydrate-related enzymes suggests an altered performance of the soil microbiome with respect to ligninolytic and cellulolytic enzymes upon long-term mitigation of drought via irrigation.

From a methodological perspective, it must be highlighted that annotation of short reads and assembled contigs provided somewhat different views on the irrigation-related shifts in gene composition. Whereas some functional groups showed the same response in the two datasets, others showed some striking differences (Figures 1, 2). This discrepancy could be attributed to the insufficient or inaccurate annotation of short reads on the one hand, or to the lack of recruitment of most sequences to the assembled contigs on the other hand, as explained in the Methods section. This observation highlights the need to perform both types of analyses to capture shifts in the composition of the metagenome and draw attention to the fact that interpretations based on only one analysis should be taken with caution.

### 4.2. Shifts in soil respiration and ^13^C-CO_2_ signatures in respired CO_2_

We hypothesized that microbial communities from irrigated soils might be more efficient in degrading lignin and cellulose than communities in dry soils. However, respiration rates in our study were neither affected by the addition of cellulose or lignin nor did they show any differences related to the long-term irrigation treatment (Supplementary Figure S4), despite the fact that the concentration of bulk soil C was lower in the irrigated soils (13%) compared to the dry soils (19%; Hartmann et al., 2017). Other SIP studies using forest soils recorded increased CO_2_ emissions up to 1 month after substrate addition (Eichorst and Kuske, 2012; Štursová et al., 2012). This might have been attributed to a lower availability of labile C in these soils compared to the forest soil used in our study.

Another picture was revealed when the ^13^C-signature in the respired CO_2_ from the degradation of the ^13^C-labeled lignin and cellulose was recorded. Incubation of the soils with ^13^C-labeled lignin and cellulose revealed respiration of both C substrates from both irrigated and dry soils (Figure 3). The higher δ^13^C values of the labelled lignin compared to the labelled cellulose samples can partly be explained by a higher total C concentration of the extracted lignin (74%) compared to the extracted cellulose (46%). Cellulose degradation is a ubiquitous trait among bacteria and fungi (Berg and McClaugherty, 2014), which might corresponds to the early start of increased δ^13^C values detected in cellulose samples. The peak at incubation day 28 might be a combination of ongoing degradation of cellulose accompanied by C recycling within the food web. Lignin has been postulated to be chemically more recalcitrant to degradation compared to cellulose, due to the linkage heterogeneity of non-phenolic and phenolic bonds (Abdel-Hamid et al., 2013). This persistence against degradation is debatable since a conceptual model for lignin degradation revealed its persisting nature only if easy degradable C sources become limited (Klotzbücher et al., 2011). In our study, lignin is degraded at a high rate and without delay, thus, contradicting the classical view of recalcitrant nature of lignin. However, some of the measured labelled CO_2_ in the lignin samples was likely derived from impurities (e.g., hemicellulose, aromatics) in the sample that cannot be avoided during lignin extraction (personal communication with IsoLife). Additionally, the lower efflux of ^13^CO_2_ from the irrigated soil at day 28 was most likely caused by limitation of C due to the lower C concentration in the irrigated soil (Hartmann et al., 2017). Nonetheless, we recorded a 2-fold increased respiration of lignin ^13^CO_2_ compared to cellulose, indicating faster utilization of lignin C irrespective of soil irrigation treatment. In contradiction to our hypothesis, the irrigated soils showed similar rates of lignin and cellulose degradation when compared to the dry soils (Figure 3) despite different taxa participating in the process (Figure 5). This might indicate some degree of functional redundancy among these microbial communities and is consistent with previous observations in this system where root litter decomposition was similar in the two treatments besides different taxa colonizing the decomposing roots over a time frame of 2 years (Herzog et al., 2019). The ability of soil microbial communities to sustain their activity under drought stress – either through physiological acclimation, shift in community composition, or evolutionary adaptation – in combination with a reduced input of plant-derived C due to a decrease in primary productivity under drought, could potentially lead to losses in soil C over time (Allison, 2023).

### 4.3. Microbial utilizers of lignin and cellulose

Little is known about lignin degrading bacteria. Here, we were able to identify five abundant groups of ASVs in the families of Sphingomonadaceae, Caulobacteraceae, Burkholderiaceae, Xanthomonadaceae and subgroup 6 of the Acidobacteria, which were enriched in the heavy fraction after ^13^C-lignin addition (Figure 6). Most genera identified in this study within the Sphingomonadaceae are known to produce ligninolytic enzymes (Tian et al., 2014; Wilhelm et al., 2019). ASVs identified as Caulobacteraceae were highly enriched in lignin samples, which is supported by other studies, where members of Caulobacteraceae family have been detected expressing ligninolytic (Danon et al., 2008; Wilhelm et al., 2019) and cellulolytic (Wilhelm et al., 2017) potential. Indeed, *Caulobacter* species appear to be abundant soil bacteria that play an important role in organic matter decomposition (Wilhelm, 2018), and have been previously shown to be enriched on lignin-amended beads and sensitive to climatic conditions such as warming (Pold et al., 2015). Xanthomonadales were another enriched group. Most members of this order are plant parasites revealing cellulolytic and pectinolytic potential (Brenner et al., 2005), and Xanthomonadaceae were reported to be highly abundant on decomposing wood (Hervé et al., 2014). However, ligninolytic capacity of this group was only detected in isolates (Zimmermann, 1990), thus, this is the first study providing evidence for significant lignin degrading activity by Xanthomonadales in soil.

Several fungal species in the Agaricomycetes, known as white-rot fungi, are well-known lignin degraders. However, surprisingly few of these fungi were enriched after lignin addition in our study (Figure 7). Enriched fungal ASVs were mainly found among the Ascomycota and a few in the white rot order Auriculariales (Basidiomycota). The order of Helotiales has been detected in fresh litter and at several decay stages in high abundance on oak tree stumps (van der Wal et al., 2015), thus, their involvement in wood degradation can only be hypothesized, since most species are known to follow an endophytic or parasitic lifestyle (Tedersoo et al., 2009). Our data revealed, uptake of lignin C by Helotiales taxa present in high abundance, suggesting saprotrophic activity after host death. ASVs of the order Hypocreales were also enriched and were mainly assigned to the genus *Ilyonectria*, a well-known plant parasite causing root rot in woody plants (Aiello et al., 2014). Another notable observation is the genus of *Mortierella*, not known for degradation of structural C compounds, although present at high abundance in decomposition studies of maple (Fisk et al., 2011) and pine roots (Li et al., 2015a). Our results demonstrate lignin C incorporation by *Mortierella* species during decomposition, and thus, help to understand its dominance on decomposing woody roots.

## 5. Conclusion

Here, we showed that mitigation of drought by a decade of irrigation stimulated profound changes in the functional capacity encoded by the soil microbiome, showing alterations in C and N metabolism as well as regulatory processes protecting from starvation and desiccation. These shifts are consistent with previous observations in this forest ecosystem, showing that the increased primary production upon irrigation promoted a soil microbiome with largely copiotrophic lifestyle and increased microbial activity. Despite the altered functional potential and the fact that different microbial taxa were involved in utilization of C from lignin and cellulose, C utilization rates from dry and irrigated soils were not clearly different, providing an indication of some functional redundancy among soil microbial communities in this system. This result is in line with previous observations in this forest ecosystem, showing that *in-situ* root litter decomposition was not markedly altered by irrigation despite different microbial taxa colonizing the decomposing roots. This potential functional redundancy in microbial decomposition under different water availabilities through community shifts might have negative consequences for soil C stocks especially if C inputs from trees decline. Our findings help to better understand the response of forest soils to increasing frequency and duration of drought, which is important to anticipate potential changes in biogeochemical cycling under future climatic conditions.

## Data availability statement

The datasets presented in this study can be found in online repositories. The names of the repository/repositories and accession number(s) can be found below: https://www.ebi.ac.uk/ena, PRJEB52100; https://www.ebi.ac.uk/ena, PRJEB22281.

## Author contributions

MH: Conceptualization, Data curation, Formal analysis, Investigation, Methodology, Software, Visualization, Writing – original draft, Writing – review and editing. CH: Conceptualization, Data curation, Formal analysis, Investigation, Methodology, Software, Visualization, Writing – original draft, Writing – review and editing. IB: Conceptualization, Funding acquisition, Investigation, Project administration, Resources, Supervision, Writing – original draft, Writing – review and editing. BS: Methodology, Writing – review and editing, Investigation. FM: Writing – review and editing, Data curation, Methodology, Software. NB: Conceptualization, Supervision, Writing – review and editing. BF: Conceptualization, Funding acquisition, Investigation, Project administration, Resources, Supervision, Writing – review and editing.

## Funding

The author(s) declare financial support was received for the research, authorship, and/or publication of this article. This study was funded by the Swiss National Science Foundation (SNF) with the grant number 31003A_149507. Open access funding by ETH Zurich.

## Conflict of interest

The authors declare that the research was conducted in the absence of any commercial or financial relationships that could be construed as a potential conflict of interest.

The author(s) declared that they were an editorial board member of Frontiers, at the time of submission. This had no impact on the peer review process and the final decision.

## Publisher’s note

All claims expressed in this article are solely those of the authors and do not necessarily represent those of their affiliated organizations, or those of the publisher, the editors and the reviewers. Any product that may be evaluated in this article, or claim that may be made by its manufacturer, is not guaranteed or endorsed by the publisher.

## Footnotes

^1^ www.rawgraphs.io
