# Supplementary material for: Long-term mitigation of drought changes the functional potential and life-strategies of the forest soil microbiome involved in organic matter decomposition
Source: Front Microbiol. 2023 Sep 29;14:1267270. doi: 10.3389/fmicb.2023.1267270 (PMC10570739; doi:10.3389/fmicb.2023.1267270)
Supplement: Supplementary file 1 [file Data_Sheet_1.PDF]

Supplementary Material

1 Supplementary Data

Supplementary Data S1: The complete list of features derived from the shotgun metagenomes annotated based on the SEED Subsystems and CAZy databases as well as the response of these features to experimental irrigation.

2 Supplementary Figures

Figure S1: Gene diversity and compositional shifts of the soil metagenomes based on annotations to the SEED Subsystems database to carbon related metabolism (level 2 categories "Carbohydrates", "Metabolism of Aromatic Compounds", and "OTHER Carbohydrates"). Left: Shannon diversity and principal coordinate ordinations (PCOs) based on the inventory of annotated genes based on short reads (top) and assembled contigs (bottom) from control (red) and irrigated (blue) forest soils. The variance explained by each PCO axis are provided in parentheses. Right: Relative changes of SEED Subsystems categories (level 2) in control (red) and irrigated (blue) plots. The horizontal bars represent z-scores (means and standard errors) of relative gene abundances indicating a proportional increase or decrease of these genes in each treatment compared to the overall mean value across the entire dataset. The effect sizes of the relative changes (z-scores) due to the treatment are provided as ANOVA F-ratios, p-values and q-values. Positive tests for heteroscedasticity (H) and non-normality of residuals (N) are flagged. Number of assigned reads across all samples in each category are provided.

Figure S2: Compositional shifts in gene composition based on annotations of the short reads to the SEED Subsystems database. Relative changes of SEED Subsystems categories from level 1 to 3 in dry (red) and irrigated (blue) plots. The horizontal bars represent z-scores (means and standard errors) of relative gene abundances indicating a proportional increase or decrease of these genes in each treatment compared to the overall mean value across the entire dataset. The effect sizes of the relative changes (z-scores) due to the treatment are provided as ANOVA F-ratios, p-values and q-values. Positive tests for heteroscedasticity (H) and non-normality of residuals (N) are flagged. Number of assigned reads across all samples in each category are provided.

Figure S3: Compositional shifts in gene composition based on annotations of the assembled contigs to the SEED Subsystems database. Relative changes of SEED Subsystems categories from level 1 to 3 in dry (red) and irrigated (blue) plots. The horizontal bars represent z-scores (means and standard errors) of relative gene abundances indicating a proportional increase or decrease of these genes in each treatment compared to the overall mean value across the entire dataset. The effect sizes of the relative changes (z-scores) due to the treatment are provided as ANOVA F-ratios, p-values and q-values. Positive tests for heteroscedasticity (H) and non-normality of residuals (N) are flagged. Number of assigned reads across all samples in each category are provided.

Figure S4: CO_2 respiration rates over the 28-day incubation period of two forest soils (control vs. irrigated) supplied with ^{13}C-labeled (13C) and non-labeled (12C) lignin (left) and cellulose (right), including the no substrate controls.

3 Supplementary Tables

Table S1: Effects of the experimental factors on the responsive fraction of the soil bacterial and fungal communities after lignin or cellulose addition as assessed by PERMANOVA (values with P<0.05 in bold).

Responsive community [1]	Lignin				Cellulose			
	Bacteria		Fungi		Bacteria		Fungi	
	F-ratio	P-value	F-ratio	P-value	F-ratio	P-value	F-ratio	P-value
FCL [2]	15.2	**<0.001**	14.1	**<0.001**	16.9	**<0.001**	12.7	**<0.001**
Treatment (T)	39.5	**<0.001**	39.7	**<0.001**	42.7	**<0.001**	52.1	**<0.001**
Day (D)	2.6	**0.012**	4.0	**<0.001**	2.7	**0.009**	5.0	**<0.001**
FCL x T	1.5	**0.030**	0.9	0.691	1.5	**0.018**	1.2	0.147
FCL x D	1.1	0.239	1.8	**0.002**	1.2	0.169	2.3	**<0.001**
T x D	0.7	0.687	0.9	0.476	0.8	0.542	0.9	0.530

[1] The responsive community consist of ASVs that showed some significant differences across fraction, C source or label.

[2] A combined factor consisting of fraction (F, heavy vs. light), C source (lignin/cellulose vs. no substrate), and label (L, ^{13}C vs. ^{12}C lignin/cellulose).

Table S2: Effects of the experimental factors on the enriched fraction of the soil bacterial and fungal communities after lignin or cellulose addition as assessed by PERMANOVA (values with P<0.05 in bold).

Enriched community [1]	Lignin				Cellulose			
	Bacteria		Fungi		Bacteria		Fungi	
	F-ratio	P-value	F-ratio	P-value	F-ratio	P-value	F-ratio	P-value
Treatment (T)	6.2	**<0.001**	6.4	**<0.001**	7.8	**<0.001**	19.4	**<0.001**
Day (D)	3.6	**<0.001**	4.7	**<0.001**	8.5	**<0.001**	9.0	**<0.001**
T x D	0.7	0.78	0.7	0.77	0.6	0.85	2.8	**0.03**

[1] The enriched community consists of ASVs that significantly increased in the heavy fraction after addition of ^{13}C-labelled lignin or cellulose.

Figure S1

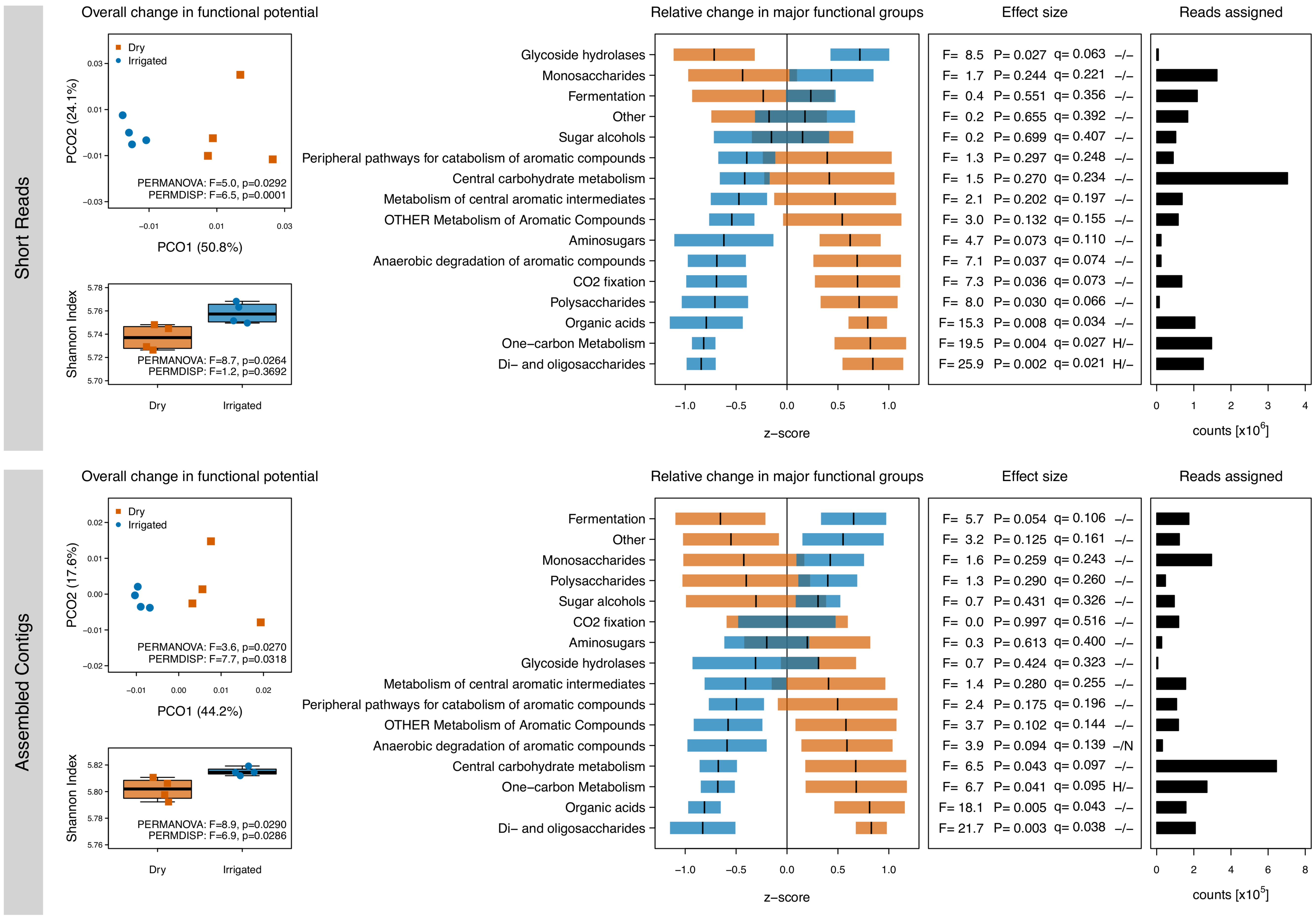

Short Reads
Overall change in functional potential
Dry
Irrigated
PCO2 (24.1%)
PCO1 (50.8%)
PERMANOVA: F=5.0, p=0.0292
PERMDISP: F=6.5, p=0.0001
Shannon Index
PERMANOVA: F=8.7, p=0.0264
PERMDISP: F=1.2, p=0.3692
Relative change in major functional groups
Glycoside hydrolases
Monosaccharides
Fermentation
Other
Sugar alcohols
Peripheral pathways for catabolism of aromatic compounds
Central carbohydrate metabolism
Metabolism of central aromatic intermediates
OTHER Metabolism of Aromatic Compounds
Aminosugars
Anaerobic degradation of aromatic compounds
CO2 fixation
Polysaccharides
Organic acids
One−carbon Metabolism
Di− and oligosaccharides
z−score
Effect size
F= 8.5 P= 0.027 q= 0.063 −/−
F= 1.7 P= 0.244 q= 0.221 −/−
F= 0.4 P= 0.551 q= 0.356 −/−
F= 0.2 P= 0.655 q= 0.392 −/−
F= 0.2 P= 0.699 q= 0.407 −/−
F= 1.3 P= 0.297 q= 0.248 −/−
F= 1.5 P= 0.270 q= 0.234 −/−
F= 2.1 P= 0.202 q= 0.197 −/−
F= 3.0 P= 0.132 q= 0.155 −/−
F= 4.7 P= 0.073 q= 0.110 −/−
F= 7.1 P= 0.037 q= 0.074 −/−
F= 7.3 P= 0.036 q= 0.073 −/−
F= 8.0 P= 0.030 q= 0.066 −/−
F= 15.3 P= 0.008 q= 0.034 −/−
F= 19.5 P= 0.004 q= 0.027 H/−
F= 25.9 P= 0.002 q= 0.021 H/−
Reads assigned
counts [x10^6]
Assembled Contigs
Overall change in functional potential
Dry
Irrigated
PCO2 (17.6%)
PCO1 (44.2%)
PERMANOVA: F=3.6, p=0.0270
PERMDISP: F=7.7, p=0.0318
Shannon Index
PERMANOVA: F=8.9, p=0.0290
PERMDISP: F=6.9, p=0.0286
Relative change in major functional groups
Fermentation
Other
Monosaccharides
Polysaccharides
Sugar alcohols
CO2 fixation
Aminosugars
Glycoside hydrolases
Metabolism of central aromatic intermediates
Peripheral pathways for catabolism of aromatic compounds
OTHER Metabolism of Aromatic Compounds
Anaerobic degradation of aromatic compounds
Central carbohydrate metabolism
One−carbon Metabolism
Organic acids
Di− and oligosaccharides
z−score
Effect size
F= 5.7 P= 0.054 q= 0.106 −/−
F= 3.2 P= 0.125 q= 0.161 −/−
F= 1.6 P= 0.259 q= 0.243 −/−
F= 1.3 P= 0.290 q= 0.260 −/−
F= 0.7 P= 0.431 q= 0.326 −/−
F= 0.0 P= 0.997 q= 0.516 −/−
F= 0.3 P= 0.613 q= 0.400 −/−
F= 0.7 P= 0.424 q= 0.323 −/−
F= 1.4 P= 0.280 q= 0.255 −/−
F= 2.4 P= 0.175 q= 0.196 −/−
F= 3.7 P= 0.102 q= 0.144 −/−
F= 3.9 P= 0.094 q= 0.139 −/N
F= 6.5 P= 0.043 q= 0.097 −/−
F= 6.7 P= 0.041 q= 0.095 H/−
F= 18.1 P= 0.005 q= 0.043 −/−
F= 21.7 P= 0.003 q= 0.038 −/−
Reads assigned
counts [x10^5]

Figure S2

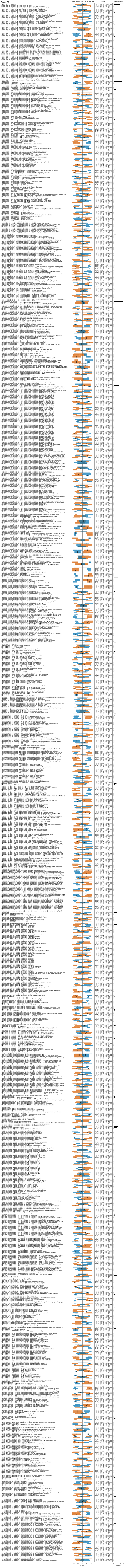
Relative change in major functional groups
Effect size
Reads assigned
L1 Amino Acids and Derivatives F= 0.1 P=0.734 q=0.417 –/–
L1 Amino Acids and Derivatives – L2 Alanine, serine, and glycine F= 0.8 P=0.416 q=0.303 –/–
L1 Amino Acids and Derivatives – L2 Alanine, serine, and glycine – L3 Alanine_biosynthesis F=100.6 P=<0.001 q=0.013 –/–
L1 Amino Acids and Derivatives – L2 Alanine, serine, and glycine – L3 Glycine_and_Serine_Utilization F= 1.2 P=0.322 q=0.260 –/–
L1 Amino Acids and Derivatives – L2 Alanine, serine, and glycine – L3 Glycine_Biosynthesis F= 0.0 P=0.916 q=0.471 –/–
L1 Amino Acids and Derivatives – L2 Alanine, serine, and glycine – L3 Glycine_cleavage_system F= 0.4 P=0.543 q=0.353 –/–
L1 Amino Acids and Derivatives – L2 Alanine, serine, and glycine – L3 Serine_Biosynthesis F= 2.9 P=0.139 q=0.159 –/–
L1 Amino Acids and Derivatives – L2 Arginine; urea cycle, polyamines F= 23.3 P=0.003 q=0.022 –/–
L1 Amino Acids and Derivatives – L2 Arginine; urea cycle, polyamines – L3 Anaerobic_Oxidative_Degradation_of_L–Ornithine F= 0.0 P=0.891 q=0.463 –/–
L1 Amino Acids and Derivatives – L2 Arginine; urea cycle, polyamines – L3 Arginine_and_Ornithine_Degradation F= 45.7 P=<0.001 q=0.014 –/–
L1 Amino Acids and Derivatives – L2 Arginine; urea cycle, polyamines – L3 Arginine_Biosynthesis_extended F= 2.6 P=0.155 q=0.170 –/–
L1 Amino Acids and Derivatives – L2 Arginine; urea cycle, polyamines – L3 Cyanophycin_Metabolism F= 0.1 P=0.819 q=0.442 –/–
L1 Amino Acids and Derivatives – L2 Arginine; urea cycle, polyamines – L3 Polyamine_Metabolism F= 18.3 P=0.005 q=0.029 –/–
L1 Amino Acids and Derivatives – L2 Arginine; urea cycle, polyamines – L3 Putrescine_utilization_pathways F= 0.0 P=0.982 q=0.486 –/–
L1 Amino Acids and Derivatives – L2 Arginine; urea cycle, polyamines – L3 Urea_carboxylase_and_Allophanate_hydrolase_clu F= 8.5 P=0.027 q=0.063 –/–
L1 Amino Acids and Derivatives – L2 Arginine; urea cycle, polyamines – L3 Urea_decomposition F= 0.7 P=0.430 q=0.310 –/–
L1 Amino Acids and Derivatives – L2 Arginine; urea cycle, polyamines – L3 Urease_subunits F= 0.4 P=0.535 q=0.350 –/–
L1 Amino Acids and Derivatives – L2 Aromatic amino acids and derivatives F= 0.1 P=0.741 q=0.419 –/–
L1 Amino Acids and Derivatives – L2 Aromatic amino acids and derivatives – L3 Aromatic_amino_acid_degradation F= 6.3 P=0.045 q=0.083 –/–
L1 Amino Acids and Derivatives – L2 Aromatic amino acids and derivatives – L3 Aromatic_amino_acid_interconversions_with_ F= 1.5 P=0.261 q=0.230 –/–
L1 Amino Acids and Derivatives – L2 Aromatic amino acids and derivatives – L3 Bacilysin_biosynthesis F= 0.5 P=0.522 q=0.346 H/–
L1 Amino Acids and Derivatives – L2 Aromatic amino acids and derivatives – L3 Chorismate_Synthesis F= 0.4 P=0.535 q=0.350 –/–
L1 Amino Acids and Derivatives – L2 Aromatic amino acids and derivatives – L3 Chorismate:_Intermediate_for_synthesis_of_ F= 0.1 P=0.770 q=0.428 –/–
L1 Amino Acids and Derivatives – L2 Aromatic amino acids and derivatives – L3 Common_Pathway_For_Synthesis_of_Aromatic_C F= 7.8 P=0.031 q=0.068 –/–
L1 Amino Acids and Derivatives – L2 Aromatic amino acids and derivatives – L3 Phenylalanine_and_Tyrosine_Branches_from_C F= 7.2 P=0.036 q=0.073 –/N
L1 Amino Acids and Derivatives – L2 Aromatic amino acids and derivatives – L3 Tryptophan_catabolism F= 11.4 P=0.015 q=0.047 –/–
L1 Amino Acids and Derivatives – L2 Aromatic amino acids and derivatives – L3 Tryptophan_synthesis F= 4.6 P=0.076 q=0.113 –/–
L1 Amino Acids and Derivatives – L2 Branched–chain amino acids F= 7.6 P=0.033 q=0.070 –/–
L1 Amino Acids and Derivatives – L2 Branched–chain amino acids – L3 Branched_chain_amino_acid_degradation_regulons F= 3.5 P=0.113 q=0.141 –/–
L1 Amino Acids and Derivatives – L2 Branched–chain amino acids – L3 Branched–Chain_Amino_Acid_Biosynthesis F= 0.0 P=0.835 q=0.447 –/–
L1 Amino Acids and Derivatives – L2 Branched–chain amino acids – L3 HMG_CoA_Synthesis F= 5.7 P=0.054 q=0.093 –/–
L1 Amino Acids and Derivatives – L2 Branched–chain amino acids – L3 Isoleucine_degradation F= 19.7 P=0.004 q=0.026 –/–
L1 Amino Acids and Derivatives – L2 Branched–chain amino acids – L3 Ketoisovalerate_oxidoreductase F= 9.4 P=0.022 q=0.056 –/–
L1 Amino Acids and Derivatives – L2 Branched–chain amino acids – L3 Leucine_Biosynthesis F= 1.7 P=0.244 q=0.221 –/–
L1 Amino Acids and Derivatives – L2 Branched–chain amino acids – L3 Leucine_Degradation_and_HMG–CoA_Metabolism F= 3.6 P=0.108 q=0.137 –/–
L1 Amino Acids and Derivatives – L2 Branched–chain amino acids – L3 Valine_degradation F= 2.6 P=0.159 q=0.172 –/–
L1 Amino Acids and Derivatives – L2 Glutamine, glutamate, aspartate, asparagine; ammonia assimilation F= 2.3 P=0.182 q=0.186 –/–
L1 Amino Acids and Derivatives – L2 Glutamine, glutamate, aspartate, asparagine; ammonia assimilation – L3 Aspartate_ami F= 15.1 P=0.008 q=0.034 –/–
L1 Amino Acids and Derivatives – L2 Glutamine, glutamate, aspartate, asparagine; ammonia assimilation – L3 Glutamate_and F= 0.0 P=0.942 q=0.477 –/N
L1 Amino Acids and Derivatives – L2 Glutamine, glutamate, aspartate, asparagine; ammonia assimilation – L3 Glutamate_deh F= 4.1 P=0.091 q=0.125 –/–
L1 Amino Acids and Derivatives – L2 Glutamine, glutamate, aspartate, asparagine; ammonia assimilation – L3 Glutamine_syn F= 3.3 P=0.118 q=0.144 –/–
L1 Amino Acids and Derivatives – L2 Glutamine, glutamate, aspartate, asparagine; ammonia assimilation – L3 Glutamine,_Gl F= 21.2 P=0.004 q=0.025 –/–
L1 Amino Acids and Derivatives – L2 Glutamine, glutamate, aspartate, asparagine; ammonia assimilation – L3 Poly–gamma–gl F= 1.2 P=0.310 q=0.254 –/N
L1 Amino Acids and Derivatives – L2 Histidine Metabolism F= 0.1 P=0.793 q=0.435 –/–
L1 Amino Acids and Derivatives – L2 Histidine Metabolism – L3 Histidine_Biosynthesis F= 0.1 P=0.792 q=0.435 –/–
L1 Amino Acids and Derivatives – L2 Histidine Metabolism – L3 Histidine_Degradation F= 0.0 P=0.966 q=0.483 H/–
L1 Amino Acids and Derivatives – L2 Lysine, threonine, methionine, and cysteine F= 0.4 P=0.557 q=0.358 –/–
L1 Amino Acids and Derivatives – L2 Lysine, threonine, methionine, and cysteine – L3 Cysteine_Biosynthesis F= 49.0 P=<0.001 q=0.014 H/–
L1 Amino Acids and Derivatives – L2 Lysine, threonine, methionine, and cysteine – L3 Lysine_biosynthesis_AAA_pathway_2 F= 0.0 P=0.914 q=0.470 –/–
L1 Amino Acids and Derivatives – L2 Lysine, threonine, methionine, and cysteine – L3 Lysine_Biosynthesis_DAP_Pathway F= 0.4 P=0.561 q=0.360 –/N
L1 Amino Acids and Derivatives – L2 Lysine, threonine, methionine, and cysteine – L3 Lysine_degradation F= 0.2 P=0.635 q=0.386 –/–
L1 Amino Acids and Derivatives – L2 Lysine, threonine, methionine, and cysteine – L3 Lysine_fermentation F= 20.8 P=0.004 q=0.025 –/–
L1 Amino Acids and Derivatives – L2 Lysine, threonine, methionine, and cysteine – L3 Methionine_Biosynthesis F= 0.9 P=0.371 q=0.284 –/–
L1 Amino Acids and Derivatives – L2 Lysine, threonine, methionine, and cysteine – L3 Methionine_Degradation F= 3.6 P=0.105 q=0.135 –/–
L1 Amino Acids and Derivatives – L2 Lysine, threonine, methionine, and cysteine – L3 Methionine_Salvage F= 0.0 P=0.858 q=0.454 –/–
L1 Amino Acids and Derivatives – L2 Lysine, threonine, methionine, and cysteine – L3 Threonine_and_Homoserine_Biosynthes F= 2.1 P=0.196 q=0.194 –/–
L1 Amino Acids and Derivatives – L2 Lysine, threonine, methionine, and cysteine – L3 Threonine_degradation F= 6.4 P=0.045 q=0.082 –/N
L1 Amino Acids and Derivatives – L2 OTHER Amino Acids and Derivatives F= 1.4 P=0.281 q=0.240 –/–
L1 Amino Acids and Derivatives – L2 OTHER Amino Acids and Derivatives – L3 Creatine_and_Creatinine_Degradation F= 4.7 P=0.073 q=0.110 –/–
L1 Amino Acids and Derivatives – L2 OTHER Amino Acids and Derivatives – L3 L–2–amino–thiazoline–4–carboxylic_acid–Lcyste F= 0.1 P=0.814 q=0.441 –/N
L1 Amino Acids and Derivatives – L2 OTHER Amino Acids and Derivatives – L3 Phosphonoalanine_utilization F= 0.6 P=0.458 q=0.322 –/–
L1 Amino Acids and Derivatives – L2 Proline and 4–hydroxyproline F= 10.1 P=0.019 q=0.053 –/–
L1 Amino Acids and Derivatives – L2 Proline and 4–hydroxyproline – L3 A_Hypothetical_Protein_Related_to_Proline_Metaboli F= 0.1 P=0.750 q=0.422 –/–
L1 Amino Acids and Derivatives – L2 Proline and 4–hydroxyproline – L3 Proline,_4–hydroxyproline_uptake_and_utilization F= 9.9 P=0.020 q=0.054 –/–
L1 Carbohydrates F= 6.2 P=0.047 q=0.084 –/–
L1 Carbohydrates – L2 Aminosugars F= 4.7 P=0.073 q=0.110 –/–
L1 Carbohydrates – L2 Aminosugars – L3 (GlcNAc)2_Catabolic_Operon F= 14.8 P=0.009 q=0.035 –/–
L1 Carbohydrates – L2 Aminosugars – L3 Chitin_and_N–acetylglucosamine_utilization F= 0.0 P=0.846 q=0.451 –/–
L1 Carbohydrates – L2 Aminosugars – L3 Fructoselysine_(Amadori_product)_utilization_pathway F= 4.9 P=0.068 q=0.106 –/–
L1 Carbohydrates – L2 Aminosugars – L3 N–Acetyl–Galactosamine_and_Galactosamine_Utilization F= 0.8 P=0.400 q=0.298 –/–
L1 Carbohydrates – L2 Aminosugars – L3 Neotrehalosadiamine_(NTD)_Biosynthesis_Operon F= 0.0 P=0.833 q=0.447 –/–
L1 Carbohydrates – L2 Central carbohydrate metabolism F= 1.5 P=0.270 q=0.234 –/–
L1 Carbohydrates – L2 Central carbohydrate metabolism – L3 Dehydrogenase_complexes F= 0.8 P=0.396 q=0.296 –/–
L1 Carbohydrates – L2 Central carbohydrate metabolism – L3 Dihydroxyacetone_kinases F= 1.9 P=0.214 q=0.204 –/N
L1 Carbohydrates – L2 Central carbohydrate metabolism – L3 Entner–Doudoroff_Pathway F= 12.6 P=0.012 q=0.042 –/N
L1 Carbohydrates – L2 Central carbohydrate metabolism – L3 Ethylmalonyl–CoA_pathway_of_C2_assimilation F= 2.1 P=0.196 q=0.194 –/–
L1 Carbohydrates – L2 Central carbohydrate metabolism – L3 Glycolate,_glyoxylate_interconversions F= 2.4 P=0.173 q=0.180 –/–
L1 Carbohydrates – L2 Central carbohydrate metabolism – L3 Glycolysis_and_Gluconeogenesis F= 0.9 P=0.380 q=0.288 –/–
L1 Carbohydrates – L2 Central carbohydrate metabolism – L3 Glycolysis_and_Gluconeogenesis,_including_Archaeal_enzymes F= 12.2 P=0.013 q=0.043 –/–
L1 Carbohydrates – L2 Central carbohydrate metabolism – L3 Glyoxylate_bypass F= 2.0 P=0.212 q=0.203 –/–
L1 Carbohydrates – L2 Central carbohydrate metabolism – L3 HPr_kinase_and_hprK_operon_in_Gram–positive_organisms F= 0.2 P=0.699 q=0.407 –/–
L1 Carbohydrates – L2 Central carbohydrate metabolism – L3 Methylglyoxal_Metabolism F= 3.4 P=0.115 q=0.143 –/–
L1 Carbohydrates – L2 Central carbohydrate metabolism – L3 Particulate_methane_monooxygenase_(pMMO) F= 4.7 P=0.073 q=0.110 –/–
L1 Carbohydrates – L2 Central carbohydrate metabolism – L3 Pentose_phosphate_pathway F= 11.8 P=0.014 q=0.045 –/–
L1 Carbohydrates – L2 Central carbohydrate metabolism – L3 Peripheral_Glucose_Catabolism_Pathways F= 0.1 P=0.774 q=0.429 –/–
L1 Carbohydrates – L2 Central carbohydrate metabolism – L3 Pyruvate_Alanine_Serine_Interconversions F= 2.9 P=0.141 q=0.161 –/–
L1 Carbohydrates – L2 Central carbohydrate metabolism – L3 Pyruvate_metabolism_I:_anaplerotic_reactions,_PEP F= 12.6 P=0.012 q=0.042 –/–
L1 Carbohydrates – L2 Central carbohydrate metabolism – L3 Pyruvate_metabolism_II:_acetyl–CoA,_acetogenesis_from_pyruvat F= 4.4 P=0.081 q=0.117 –/–
L1 Carbohydrates – L2 Central carbohydrate metabolism – L3 Pyruvate:ferredoxin_oxidoreductase F= 0.3 P=0.588 q=0.369 –/–
L1 Carbohydrates – L2 Central carbohydrate metabolism – L3 Soluble_methane_monooxygenase_(sMMO) F= 46.8 P=<0.001 q=0.014 –/–
L1 Carbohydrates – L2 Central carbohydrate metabolism – L3 TCA_Cycle F= 0.3 P=0.577 q=0.365 –/–
L1 Carbohydrates – L2 CO2 fixation F= 7.3 P=0.036 q=0.073 –/–

Figure S5

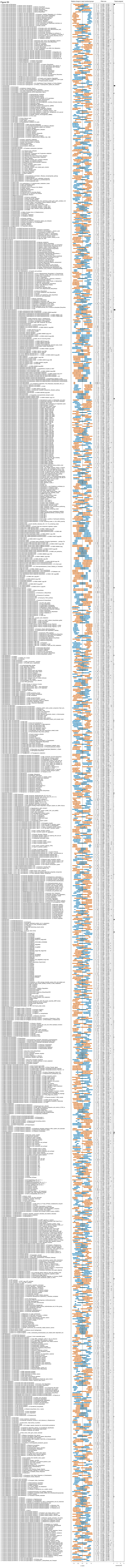

Figure S4

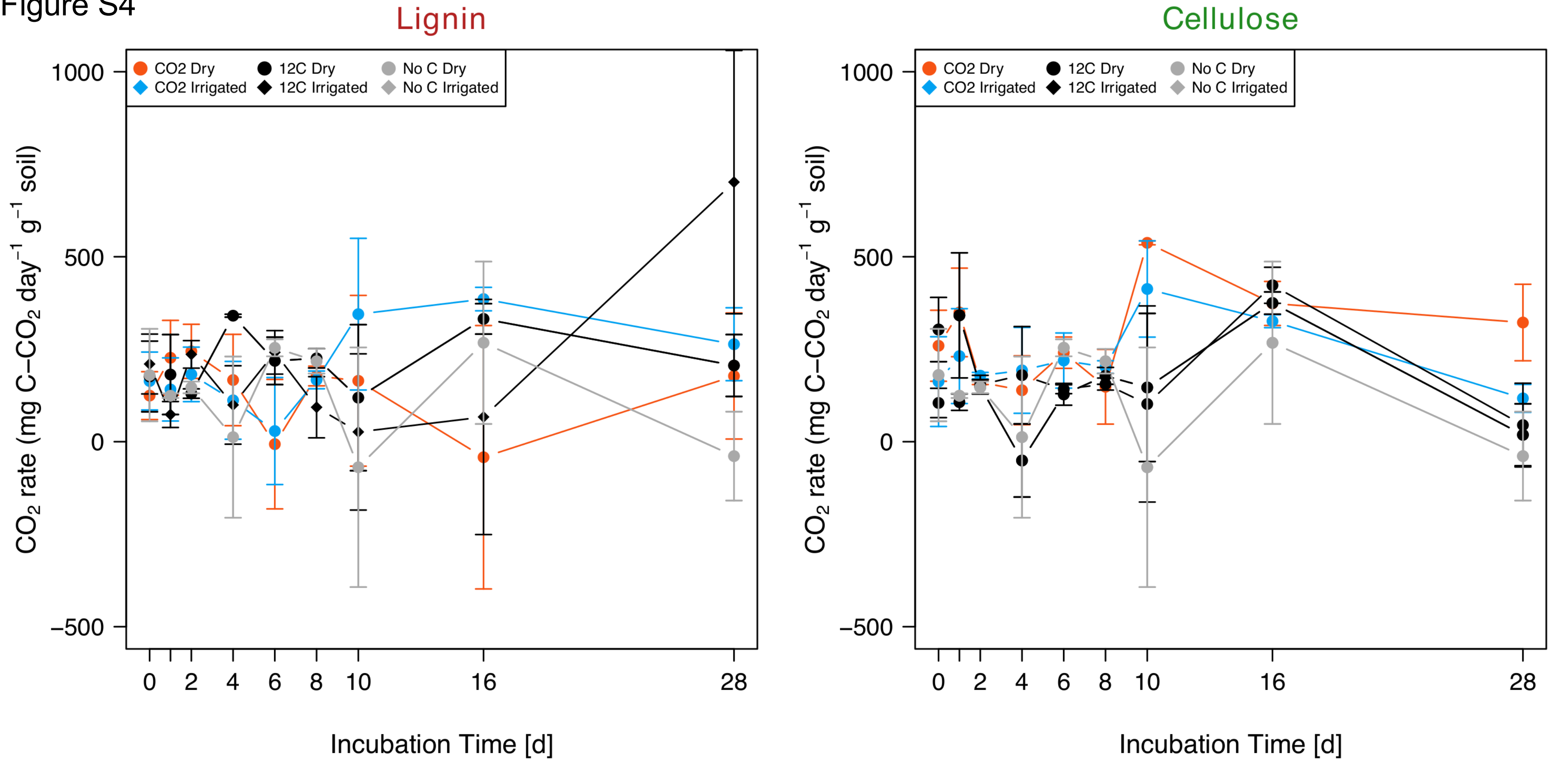

Lignin
Cellulose
CO2 Dry
CO2 Irrigated
12C Dry
12C Irrigated
No C Dry
No C Irrigated
CO2 rate (mg C–CO2 day−1 g−1 soil)
1000
500
0
−500
0
2
4
6
8
10
16
28
Incubation Time [d]
